# Mortality risk associated with heatwave exposure based on daily maximum, minimum, and combined temperature thresholds

**DOI:** 10.1093/eurpub/ckaf199

**Published:** 2025-11-06

**Authors:** Zhouxin Yin, Leyuan Xiao, Cheng Tang, Shihan Zhen, Qian Li, Yan Dou, Zhiyi Xiao, Fengchao Liang, Xiaohua Liang

**Affiliations:** School of Public Health and Emergency Management, School of Medicine, Southern University of Science and Technology, Shenzhen, China; Department of Clinical Epidemiology and Biostatistics, Children’s Hospital of Chongqing Medical University, National Clinical Research Center for Child Health and Disorders, Ministry of Education Key Laboratory of Child Development and Disorders, Chongqing Key Laboratory of Pediatrics, Chongqing Municipal Health Commission Key Laboratory of Children’s Vital Organ Development and Diseases, Chongqing, China; Center for Disease Control and Prevention of Jiulongpo District, Chongqing, China; School of Public Health and Emergency Management, School of Medicine, Southern University of Science and Technology, Shenzhen, China; School of Public Health and Emergency Management, School of Medicine, Southern University of Science and Technology, Shenzhen, China; Department of Epidemiology, Fuwai Hospital, National Center for Cardiovascular Diseases, Chinese Academy of Medical Sciences and Peking Union Medical College, Beijing, China; School of Public Health and Emergency Management, School of Medicine, Southern University of Science and Technology, Shenzhen, China; School of Public Health and Emergency Management, School of Medicine, Southern University of Science and Technology, Shenzhen, China; Department of Clinical Epidemiology and Biostatistics, Children’s Hospital of Chongqing Medical University, National Clinical Research Center for Child Health and Disorders, Ministry of Education Key Laboratory of Child Development and Disorders, Chongqing Key Laboratory of Pediatrics, Chongqing Municipal Health Commission Key Laboratory of Children’s Vital Organ Development and Diseases, Chongqing, China

## Abstract

Most prior studies assessed heatwave-related mortality using daily mean temperature as an indicator, limiting the ability to differentiate between daytime and nighttime heat effects. We collected individual mortality records with corresponding residential exposure data on daily temperature, relative humidity and ozone during warm seasons from 2016 to 2022 in Jiulongpo district, Chongqing, China. Heatwaves were categorized into three types: those defined by daily maximum temperature, daily minimum temperature, and a combination of both. A time-stratified case-crossover design was applied to assess the associations between heatwaves and mortality. During the study period, 17 552 deaths were recorded. We observed that heatwaves defined by combined temperature thresholds were associated with the highest mortality risks, with odds ratios (ORs) ranging from 1.08 (95% CI: 1.01–1.15) to 1.32 (95% CI: 1.17–1.48) under different heatwave definitions. For heatwaves defined by daily maximum temperature, ORs ranged from 1.06 (95% CI: 0.98–1.14) to 1.13 (95% CI: 1.03–1.24), while heatwaves defined by daily minimum temperature showed ORs ranging from 1.04 (95% CI: 0.99–1.10) to 1.27 (95% CI: 1.13–1.43). Exposure to heatwaves was consistently associated with increased risks of all-cause and cardiovascular mortality, but not respiratory mortality. The associations were stronger among men and under higher ozone conditions compared to their counterparts. Exposure to heatwaves significantly increased mortality risks, with the highest risks observed for compound heatwaves involving both daytime and nighttime heat. These findings underscore that the health risks associated with nighttime heat exposure should not be overlooked.

## Introduction

Climate change is projected to increase extreme temperature events in both frequency and intensity, remaining a major health challenge in the 21st century [1]. A country-wide modeling study estimated that China experienced ∼593 900 temperature-related deaths in 2019, of which 13.9 thousand could be attributable to high temperatures [2]. Heatwaves are defined as multiple, consecutive, high-temperature days [3], considered one of the most lethal weather phenomena. Several prior studies have investigated the associations between heatwave exposure and mortality outcomes by applying daily mean temperatures as an indicator [3–5]. However, this univariate definition is insufficient to characterize the difference between daytime and nighttime heatwaves fully. Evidence suggests that daytime and nighttime high temperatures differentially affect morbidity and mortality [6], and continuous hot days and nights may intensify thermoregulation strain, increasing health risks. Therefore, compound heatwave, which combines continuous scorching days and sweltering nights, is deemed to cause unprecedented health hazards given that it is projected to become a more normal situation in the future [7]. This tremendous rising trend calls for research to investigate the potential health effects of compound heatwaves, but there are to date few studies to examine the mortality impacts.

Meanwhile, in the current context of global warming, frequent high temperatures are expected to increase the reactivity of atmospheric photochemical reactions and thereby associated with elevated surface ozone concentration, leading to concurrent events of high ozone pollution and heatwaves [8], which could trigger combined negative health effects on populations through common biological pathways and thus make the synergistic effect possible [9, 10]. Besides, several studies found both low and high relative humidity could affect the heat-related mortality risks [11, 12]. To date, little such evidence was available, largely hindering the full understanding of the adverse effects of multiple environmental exposures and preventing individuals from taking targeted protection.

Chongqing, the largest megacity in southwest China, is a high-density mountainous city and thus is more seriously affected by heatwaves due to its special topography and urban morphology. We conducted an individual-level case-crossover study from 2016 to 2022 in Jiulongpo district, Chongqing, China, aiming to quantify the mortality risk of exposure to heatwaves of different types. We investigated cause-specific mortality and also assessed the potential effect modifications of sex, age, ozone, and relative humidity level.

## Methods

### Study population

During the study period of 2016–2022, mortality data with individual records, including sex, age, race, date of death, cause of death, and residential address, were extracted from the mortality surveillance system in Jiulongpo district, Chongqing, China. Subjects who did not live in Chongqing permanently before death and those with missing information were excluded. The cause of death was coded based on the International Classification of Diseases-10th revision (ICD-10) and classified into all causes (codes: A00-Z99), cardiovascular diseases (CVD, codes: I00-I99), and respiratory diseases (codes: J00-J98). To explore the effects on disease subtypes and ensure statistical efficiency at the same time, we further divided CVD cases into coronary heart disease (CHD, codes: I20-I25), stroke (codes: I60-I69), hemorrhagic stroke (codes: I60-I61), and ischemic stroke (codes: I63), and investigated chronic obstructive pulmonary disease (COPD, codes: J41-J44) separately from respiratory diseases.

### Exposure assessment

Daily maximum, minimum, and mean temperature, and mean relative humidity with a resolution of 0.1° × 0.1° were extracted from meteorological datasets developed by the European Center for Medium-Range Weather Forecasts (https://cds.climate.copernicus.eu, latest accessed on 11 October 2025). To dilute the potential bias from the coarse resolution and consider people’s activity space, we used the inverse distance weighting (IDW) method to interpolate spatial data to 1 km × 1 km. We then assigned estimates to each case according to their home addresses and interest of days.

Hourly ambient air pollution data, including fine particulate matter (PM_2.5_), inhalable particulate matter (PM_10_), ozone (O_3_), nitrogen dioxide (NO_2_), sulfur dioxide (SO_2_), and carbon monoxide (CO), at fixed-site stations were collected from the China National Urban Air Quality Real-time Publishing Platform (https://air.cnemc.cn : 18007/, latest accessed on 11 October 2025), which is administered by the China National Environmental Monitoring Centre. Following prior studies, daily maximum 8-hour average ozone concentrations were calculated and used as the main exposure metric. We applied the IDW method to assess an individual’s ozone exposure by computing the weighted average of measurements from the four most neighboring stations, in which the weight was inversely proportional to the square of the distance between each neighboring station and the residential addresses of each death case.

### Heatwave definition

In accordance with prior studies on extreme temperature events [3–5], we defined a heatwave as a period with high temperatures exceeding a certain threshold for several consecutive days. Considering the climatic condition and population acclimatization, relative thresholds (90th, 92.5th, 95th, and 97.5th percentile) were used based on the distribution of daily temperature from 2016 to 2022 in Jiulongpo district, Chongqing. We categorized heatwaves into three distinct types based on the temperature indicator used [7, 13], i.e. heatwaves based on daily maximum temperature, heatwaves based on daily minimum temperature, and compound heatwaves, defined by exceedance of both maximum and minimum temperature thresholds. These types represent daytime heatwaves, nighttime heatwaves, and day-and-night heatwaves, respectively. At the same time, the duration of heatwaves was defined as 2 or more consecutive days, 3 or more consecutive days, and 4 or more consecutive days, respectively. In total, we made three types of heatwaves, each comprising 12 definitions (Supplementary Table S1). These varying thresholds and durations allowed us to indirectly assess the influence of heatwave intensity and duration on mortality risk. The days matched with each death case were divided into heatwave or non-heatwave days according to the definitions.

### Study design

A time-stratified case-crossover design was used to assess the association of exposure to heatwaves of different types with mortality, which is widely applied in environmental epidemiology studies [14]. For each subject, the case day is defined as the date of death, and the control days come from the same day of the other weeks in the same month and year to control the potential confounding effects of day of week, long-term trend, and seasonality [15]. This approach is grounded in the counterfactual framework, which assumes that control days represent the hypothetical exposure the subject would have experienced if death had not occurred [14]. In this case, 3–4 control days were selected for each case day. Since each case serves as its own control, this design is able to effectively control factors that do not change over time in the short term, such as age, sex, race, ethnicity, socioeconomic status, chronic co-disease, and other lifestyle risk factors. The same subject’s exposure to heatwave events on case day was compared with their counterfactual exposure on control days to assess the impact of heatwave exposure on mortality.

### Statistical analyses

Our analyses were restricted to the warm season, defined as May to September, given that heatwaves mainly occurred in these months. The heatwave was included in the model as a binary variable and a conditional logistic regression model was used to separately evaluate the associations between heatwaves of different types and the risks of mortality [16]. To eliminate the potential confounding effects of other environmental factors, we adjusted for ambient ozone using a 4-day moving average from lag 0 to lag 3 (lag03), modeled with a natural cubic spline with 3 degrees of freedom (dfs), consistent with previous studies [17]. Current-day relative humidity (lag 0) was included as a linear term, as exploratory analyses indicated an approximately linear relationship with mortality risk in our study. Public holidays were also adjusted for as a binary variable. The odds ratio (OR) and its 95% confidence interval (CI) were reported to quantify the mortality effect of heatwave exposure. In order to identify susceptible populations, we conducted subgroup analyses from the perspectives of sex (man versus woman) and age (<75 years versus ≥75 years). The age stratification conforms to the age distribution of the study population (median age, 76.0 years). We divided ozone and relative humidity levels into low and high exposure according to the World Health Organization’s guidance level (100 μg/m^3^) and 70% relative humidity, which roughly corresponded to the mean value of humidity distribution, respectively. The significance of the difference between the two subgroups was evaluated by a two-sample *Z*-test [18].

We conducted several sensitivity analyses to test the robustness of our findings. First, we redefined heatwaves using daily mean temperature instead of daily maximum or minimum. We adjusted for co-exposure to PM_2_._5_, PM_10_, NO_2_, SO_2_, and CO to account for air pollution confounding. Additionally, to evaluate potential lagged effects of heatwaves, we incorporated lag structures (e.g. lag 0–3 days) in alternative models. Cumulative effects were assessed using the frequency of heatwave days, with a greater number of heatwave days within the lag window indicating stronger impacts. To test referent selection, we applied a symmetric bidirectional approach using control days from 14 days before and after the case day. We also replaced the linear term of relative humidity with a natural cubic spline (2 dfs) to allow for non-linearity. Lastly, we excluded data from 2020 and 2021 to evaluate potential impacts of the COVID-19 pandemic. All our data processing and statistical analysis were done using R version 4.2.2 (R Foundation for Statistical Computing, Vienna, Austria), and a two-sided test with a *P* values <.05 was considered to be statistically significant.

## Results

The distributions of total mortality cases and the number of days experiencing heatwaves based on daily maximum temperature, daily minimum temperature, and compound heatwaves were illustrated in Fig. S1. Table 1 summarizes the characteristics of a total of 17 552 death cases analysed in our study, among which 6819 (38.9%), 3391 (19.3%), 2458 (14.0%), 943 (5.4%), 935 (5.3%), 2614 (14.9%), and 1357 (7.7%) died from cardiovascular diseases, coronary heart disease, stroke, hemorrhagic stroke, ischemic stroke, respiratory diseases, and chronic obstructive pulmonary disease, respectively. Of all subjects, 10 430 (59.4%) were men, and 8150 (46.4%) died before 75 years.

**Table 1.** Basic characteristics of the study population from 2016 to 2022^a^.

Characteristic	*N* (%)
No. deaths (case days)	17,552
No. control days	60,131
Sex, *n* (%)	
Man	10,430 (59.4)
Woman	7120 (40.6)
Unknown	2 (0)
Age at death, years	
Mean (SD)	73.4 (14.9)
Median (IQR)^b^	76.0 (19.0)
<75, *n* (%)	8150 (46.4)
≥75, *n* (%)	9336 (53.2)
Unknown, *n* (%)	66 (0.4)
Cause of death	
Cardiovascular diseases	6819 (38.9)
Coronary heart disease	3391 (19.3)
Stroke	2458 (14.0)
Hemorrhagic stroke	943 (5.4)
Ischemic stroke	935 (5.3)
Respiratory diseases	2614 (14.9)
Chronic obstructive pulmonary disease	1357 (7.7)
Year at death, *n* (%)	
2016	2733 (15.6)
2017	2519 (14.4)
2018	2270 (12.9)
2019	2502 (14.3)
2020	2693 (15.3)
2021	2305 (13.1)
2022	2530 (14.4)

a With the rounding-off method used, the sum of the proportions may not be equal to 100%.

b Abbreviations: IQR, inter-quartile range; SD, standard deviation.

The heatwave exposure of the study population on case days and control days is shown in Supplementary Table S1. And 60 131 control days were selected for 17 552 deaths. In general, the proportion of heatwave days to total case days was larger than that on control days. The number of heatwave days varied slightly across the three definitions. The number exhibited a decreasing tendency with increasing temperature thresholds and consecutive days, ranging from 3352 (19.1%) with the definition of P90_2d to 652 (3.7%) with the definition of P97.5_4d for compound heatwaves on case days. The average exposure to daily maximum 8-hour mean ozone, daily maximum temperature, daily minimum temperature and relative humidity was 108.9 μg/m^3^, 30.2°C, 23.1°C, and 74.8%, respectively (Supplementary Table S2).

**Figure 1.**
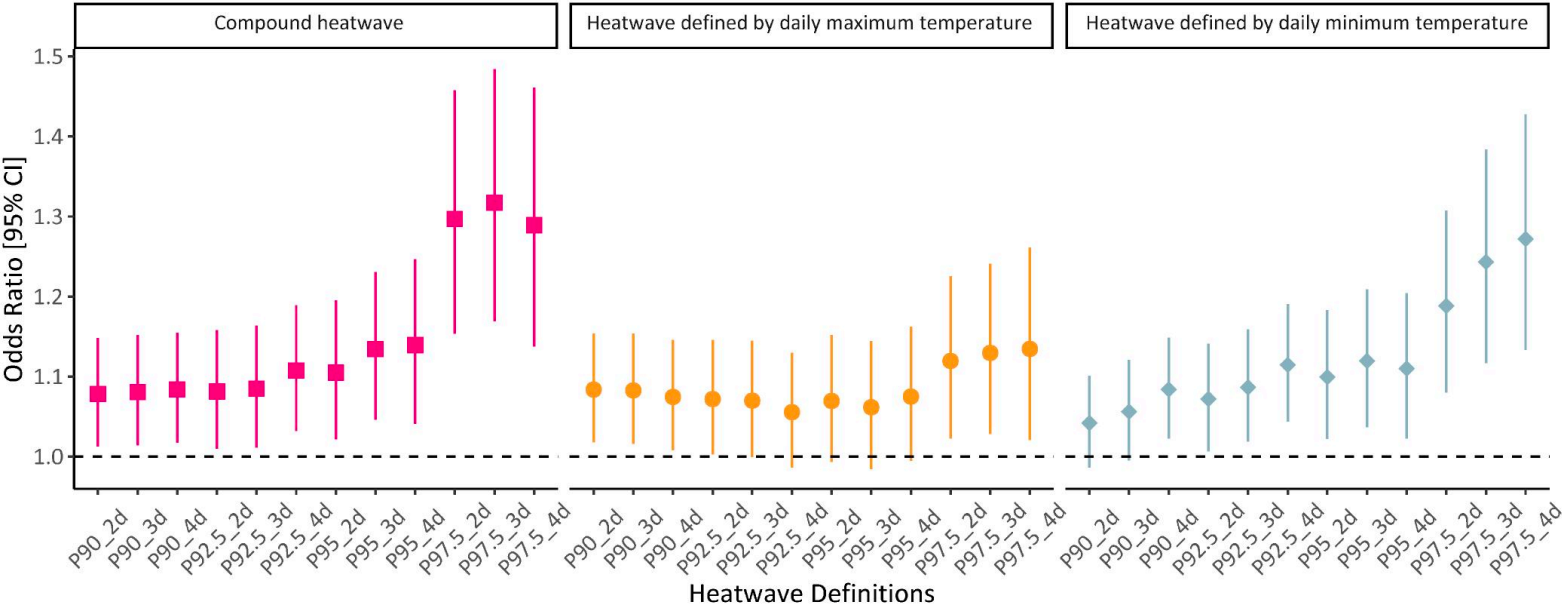
Odds ratios (with 95% CIs) for mortality associated with heatwaves across different definitions.

Figure 1 depicts the OR and 95% CI of mortality associated with heatwaves across different definitions. The effect estimates for compound heatwaves and heatwaves based on daily minimum temperature become gradually higher with thresholds rising and duration lasting, increasing from 1.08 [95% confidence interval (CI): 1.01–1.15] with the definition of P90_2 to 1.29 (95% CI: 1.14–1.46) with the definition of P97.5_4 for compound heatwaves, and from 1.04 (95% CI: 0.99–1.10) to 1.27 (95% CI: 1.13–1.43) for heatwaves based on daily minimum temperature (Supplementary Table S3). In contrast, the associations between heatwave exposure defined by daily maximum temperature and mortality were generally steady, ranging from 1.08 (95% CI: 1.02–1.15) to 1.13 (95% CI: 1.02–1.26). When the heatwave threshold reached the 97.5th percentile, the odds ratios for heatwaves defined by daily minimum temperature and for compound heatwaves appeared substantially higher than those based on daily maximum temperature under the same definition. Sensitivity analyses showed that the estimates were largely robust when using daily mean temperature as the temperature metric or further adjusting for the particulate matter and gaseous pollutants in our main analyses (Supplementary Table S4–S9). When lagged effects were considered, we found that the heatwave-related mortality risks were strong on the day of exposure and began to decline after 2 days (Supplementary Table S10). The cumulative impact, assessed as the number of heatwave days within lag 0–3, showed higher mortality risks with increasing frequency, exceeding the single-day effects (Supplementary Table S11). The results were largely consistent with those from the time-stratified design, as shown in Supplementary Table S12, suggesting that our conclusions are robust to the choice of control day selection strategy. Despite reduced statistical significance for some associations under the flexible modeling of relative humidity (Supplementary Table S13), stronger associations remained for heatwaves with higher thresholds, longer durations, and those defined by minimum and combined temperature thresholds. Finally, excluding the pandemic years (2020 and 2021) did not materially alter the associations (Supplementary Table S14), suggesting that our findings are unlikely to be substantially influenced by COVID-19-related disruptions.

**Figure 2.**
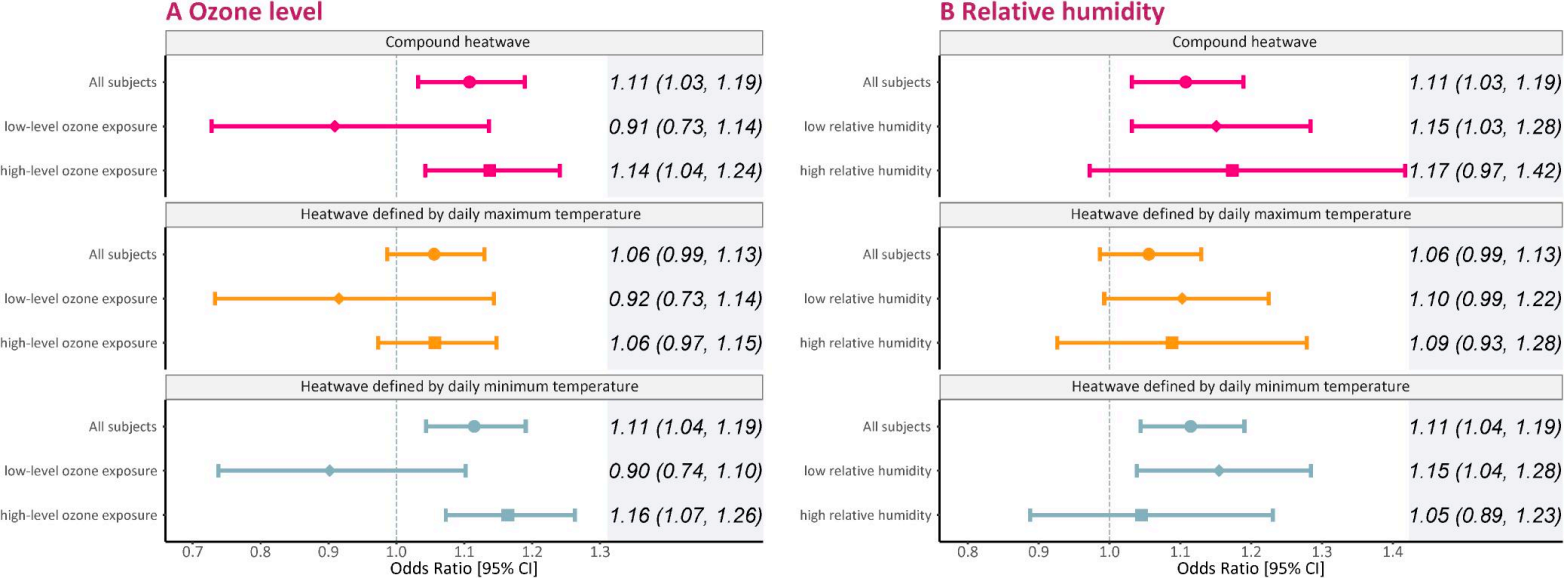
Odds ratios of mortality associated with heatwaves of different time types at different ambient ozone (A) and relative humidity (B) levels. The definition of heatwave was P92.5_4d. Abbreviation: CI, confidence interval.

As seen in Fig. 2, stronger mortality risks were observed among subjects with higher ozone pollution compared to those in lower ozone groups. A positive modification effect of ozone on heatwave-related mortality was detected for heatwaves defined by daily minimum temperature (*P* for difference: .02). Regarding relative humidity, although no statistically significant interaction was detected, heatwave-related mortality effects were only significant in the low humidity group.

Table 2 displays the subgroup-specific associations of mortality with heatwave exposure under the definition of P92.5_4d, stratified by sex, age, and cause of death. The heatwave effect on mortality did not vary by age. Larger effect estimates of heatwave exposure on mortality were observed among men, although significant differences were only identified in heatwaves defined by daily minimum temperature (*P* for difference: .03). In terms of death causes, we observed that the effects of heatwave exposure were more evident in CHD mortality than in other causes. The excess risks of mortality from CHD were almost double to triple those from all causes, corresponding to 35% (OR: 1.35; 95% CI: 1.15–1.58), 27% (OR: 1.27; 95% CI: 1.09–1.48), and 35% (OR: 1.35; 95% CI: 1.17–1.57) for compound heatwaves, heatwaves defined by daily maximum temperature, and heatwaves defined by daily minimum temperature, respectively. The impacts on stroke and respiratory diseases were generally statistically insignificant. Results for alternative heatwave definitions (P90_2d to P92.5_4d) are presented in Tables S15 to S17, showing patterns consistent with the main subgroup analyses, while more extreme thresholds (P95 and P97.5) were not shown due to limited events and less stable estimates.

**Table 2.** Odds ratios of mortality associated with heatwaves of different time types, stratified by sex, age, and cause of death^a^.

Subgroup	OR (95% CI)		
	Compound heatwave	Heatwave defined by daily maximum temperature	Heatwave defined by daily minimum temperature
Sex			
Man	1.17 (1.07–1.28)	1.09 (1.00–1.19)	1.18 (1.09–1.29)
Woman	1.02 (0.91–1.14)	1.01 (0.91–1.12)	1.02 (0.92–1.13)
*P* value^b^	.06	.26	.03
Age, y			
<75	1.14 (1.03–1.26)	1.05 (0.95–1.16)	1.14 (1.04–1.26)
≥75	1.08 (0.98–1.19)	1.06 (0.96–1.16)	1.09 (0.99–1.19)
*P* value^b^	.44	.97	.46
Cause of death			
Cardiovascular diseases	1.15 (1.03–1.29)	1.09 (0.97–1.21)	1.17 (1.05–1.30)
Coronary heart disease	1.35 (1.15–1.58)	1.27 (1.09–1.48)	1.35 (1.17–1.57)
Stroke	0.88 (0.73–1.06)	0.81 (0.68–0.97)	0.92 (0.77–1.09)
Hemorrhagic stroke	0.89 (0.66–1.20)	0.91 (0.68–1.21)	0.89 (0.67–1.17)
Ischemic stroke	1.05 (0.78–1.42)	0.95 (0.71–1.27)	1.07 (0.80–1.42)
Respiratory diseases	1.07 (0.89–1.28)	1.02 (0.86–1.21)	1.04 (0.88–1.24)
COPD^c^	1.14 (0.89–1.47)	1.12 (0.88–1.42)	1.06 (0.83–1.34)

a The definition of heatwave was P92.5_4d.

b Estimated by the two-sample Z-test.

c Abbreviations: CI, confidence interval; COPD, chronic obstructive pulmonary disease; OR, odds ratio.

## Discussion

In this individual-level case-crossover study in Chongqing, China, we found robust evidence that exposure to heatwaves with different types was associated with higher risks of mortality, and the risks of compound heatwaves were more pronounced than those occurring only in the daytime or nighttime. The findings might have important implications for developing a heat warning system and alleviating the mortality burden associated with heatwave exposure.

Significant associations between heatwave exposure and mortality are well-documented and largely consistent throughout the literature [3–5, 19, 20]. A recent meta-analysis [19] confirmed increased cardiovascular mortality risks with greater heatwave intensity, aligning with our findings. Exposure to high temperature can increase sweating and skin blood flow, straining the cardiovascular system [21]. Thus, the higher the intensity or longer the duration of a heatwave, the larger loads are exerted on the cardiovascular system and the greater the health impacts [5]. Our results also indicated that the health risks of heatwaves may extend into the 2–3 days following exposure, underscoring the importance of maintaining protective measures even after the heatwave has passed. A study of the 2017 heatwaves in 91 Chinese counties [22] estimated 16 299 excess deaths and 61.3 billion RMB in economic losses. These findings underscore the urgent need for great attention to heatwaves in a warming climate.

Prior studies have generally applied daily mean temperature to investigate heatwave-related mortality [3–5], but few have differentiated between daytime and nighttime heatwaves, which could induce adverse effects through distinct pathophysiological mechanisms. Our study uniquely classifies traditional heatwaves into three types and finds higher mortality risks associated with compound heatwaves and those defined by daily minimum temperature. While daytime heat can cause dehydration, increased blood viscosity, and cardiac overload [23], nighttime heat may disrupt circadian rhythms and impair cardiovascular recovery during sleep [24]. These effects may intensify under extreme conditions, as we observed that at higher heatwave intensities, mortality risks estimated using daily minimum temperature were more similar to those from the combined definition, suggesting that nighttime thermal stress may dominate health risks when extreme heat persists. Without adequate nighttime relief, the body’s ability to recover is compromised, leading to cumulative thermal stress [25]. Only taking the effects resulting from hot days into account would underestimate the disease burden caused by extreme temperatures. Given that a large population will be exposed to unprecedented compound heatwaves, more relative evidence is urgently needed to develop adaptation strategies.

In a global warming context, the occurrence of heat and ozone exposure becomes more and more frequent. Many studies have documented that ozone pollution can modify the mortality effects of high temperatures [26, 27], while studies focusing on effect modification by ozone for heatwave-related mortality remain elusive [28, 29]. A meta-analysis calculated pooled heatwave-related mortality at low and high ozone levels and failed to find a significant modification effect [26]. To address the knowledge gaps, two case-crossover studies in 250 Chinese cities and Jiangsu Province explored the interactions between heatwave and ambient ozone and reported that the association between heatwave exposure and mortality was stronger at higher ozone exposure levels recently [17, 30]. Our research has found similar results, and indicates that coincident heatwaves and ozone pollution at night were also very harmful. Heatwave exposure and ozone pollution may cause adverse impacts through common biological pathways, including systemic inflammation and oxidative stress, making individual effects enhanced. In addition, we found that heatwave-related mortality risks were only significant at lower relative humidity levels. While previous studies have reported elevated risks under both hot-humid and hot-dry conditions due to impaired thermoregulation [11, 12], our analysis in Chongqing revealed relatively higher risks in drier environments. This may be partially explained by the regional climatic context. In Chongqing, heatwaves are often more intense and prolonged when accompanied by lower humidity levels, potentially exacerbating dehydration and circulatory strain [31, 32]. Given that Chongqing generally experiences high average relative humidity, typically ranging from 75% to 85%, and exceeding 85% in autumn, such dry-heat events may represent particularly extreme conditions for the local population. The impact of humidity should therefore be interpreted with caution, and future studies across diverse climatic regions using interaction or joint models are warranted to better disentangle its complex interplay with heat on health outcomes. Intervention and adaptation policies should be strengthened, with special attention to the interaction between heat and other extreme environmental events, which will become an important part of in-depth research on the relationship between the environment and health.

Similar to prior studies [33, 34], our results showed that men have a higher vulnerability to heat-related mortality risk than women, which may be related to the fact that they spend more time working outdoors [35]. Contrary to current understanding, we observed that individuals under 75 years old experienced a slightly higher risk than those over 75, though the differences were not statistically significant. This may reflect better adaptive behaviors or interventions among older adults [36], such as increased awareness, air conditioning use, and health monitoring. Nevertheless, exposure misclassification or survival bias cannot be ruled out. A recent cohort study in older Chinese adults similarly found no clear age-risk gradient [37]. Rather, the study highlighted that functional aging indicators, such as limitations in mobility, activities of daily living (ADL), and cognitive function, were more predictive of vulnerability. Future studies employing cohort designs with individual-level data on function, behavior, and environment are warranted to further elucidate these complex vulnerabilities.

We estimated greater impacts of heatwaves on cardiovascular mortality, particularly coronary heart disease, than on total and respiratory mortality. Our results suggested strong evidence of an excess risk of heatwaves on CHD mortality, almost double to triple those from all causes. Subjects with CHD may bear an increased load on the circulation system to maintain normal temperature, raising the susceptibility to heatwaves. This finding is similar to previous studies [3, 38], which observed higher impacts of heatwaves on cardiopulmonary disease mortality. However, we did not find significant associations between heatwave exposure and stroke and respiratory mortality, which may be due to a small sample size of stroke and respiratory deaths in our study and thus limited ability to detect tenuous associations. More large-scale epidemiological studies are needed to clarify this issue further.

Some limitations have to be elucidated. First, we used exposure concentrations at individuals’ residences for the proxy of personal exposure, which may cause some exposure misclassification. Nonetheless, the misclassification is generally non-differential and thus may bias our estimates toward null [39]. Second, although the case-crossover design could inherently control for time-invariant confounders in a short time, we cannot exclude the possibility that the estimates were biased by residual confounding. Third, although we applied multiple heatwave definitions to reflect variation in intensity and duration, the independent and interactive effects were not explicitly modeled. Finally, as the study was limited to one district in Chongqing, generalizability should be approached with caution.

## Conclusions

In summary, this individual-level case-crossover study in Chongqing, China suggested positive associations between exposure to heatwaves of different types, particularly compound heatwaves and heatwaves based on daily minimum temperature, and increased risks of mortality. Men, people with coronary heart disease, and those exposed to higher ozone concentrations appeared more vulnerable. The findings support targeted public health interventions and underscore the urgency of strengthening heatwave protection measures.

## Supplementary Material

ckaf199_Supplementary_Data

## Data Availability

The data underlying this article cannot be shared publicly due to privacy and confidentiality restrictions involving individual participants. The data will be made available on reasonable request to the corresponding author.
