# Supplementary material for: Mortality risk associated with heatwave exposure based on daily maximum, minimum, and combined temperature thresholds
Source: Eur J Public Health. 2025 Nov 6;36(2):ckaf199. doi: 10.1093/eurpub/ckaf199 (PMC13017723; doi:10.1093/eurpub/ckaf199)
Supplement: ckaf199_Supplementary_Data [file ckaf199_supplementary_data.docx]

**Supplemental Materials**

**Mortality Risk Associated with Heatwaves Exposure Based on Daily Maximum, Minimum, and Combined Temperature Thresholds**

Zhouxin Yin ^a, #^, Leyuan Xiao ^b, #^, Cheng Tang ^c^, Shihan Zhen ^a^, Qian Li ^a^, Yan Dou ^d^, Zhiyi Xiao ^a^, Fengchao Liang ^a, *^, Xiaohua Liang ^b, *^

^a^ School of Public Health and Emergency Management, Southern University of Science and Technology, Shenzhen 518055, China.

^b^ Department of Clinical Epidemiology and Biostatistics, Children’s Hospital of Chongqing Medical University, National Clinical Research Center for Child Health and Disorders, Ministry of Education Key Laboratory of Child Development and Disorders, Chongqing Key Laboratory of Pediatrics, Chongqing Municipal Health Commission Key Laboratory of Children's Vital Organ Development and Diseases, Chongqing 400016, China.

^c^ Center for Disease Control and Prevention of Jiulongpo District, Chongqing 400039, China

^d^ Department of Epidemiology, National Center for Cardiovascular Diseases, Fuwai Hospital, Key Laboratory of Cardiovascular Epidemiology, Chinese Academy of Medical Sciences, Chinese Academy of Medical Sciences and Peking Union Medical College, Beijing 10037, China.

^#^ Zhouxin Yin and Leyuan Xiao have the same contribution.

^*^ Corresponding author: Prof. Fengchao Liang, School of Public Health and Emergency Management, Southern University of Science and Technology, Shenzhen 518055, China. E-mail: liangfc@sustech.edu.cn

and Prof. Xiaohua Liang, Department of Clinical Epidemiology and Biostatistics, Children’s Hospital of Chongqing Medical University, National Clinical Research Center for Child Health and Disorders, Ministry of Education Key Laboratory of Child Development and Disorders, Chongqing Key Laboratory of Pediatrics, Chongqing 400016, China. E-mail: xiaohualiang@hospital.cqmu.edu.cn.

**Table of Contents**

**Figure S1.** Spatial distribution of (A) death cases, (B) compound heatwave, (C) heatwave defined by daily maximum temperature, and (D) heatwave defined by daily minimum temperature from 2016 to 2022.

**Table S1.** Descriptive statistics of heatwave definitions and number of heatwave days on case days and control days from 2016 to 2022.

**Table S2.** Summary statistics of ozone and meteorological variables.

**Table S3.** Odds ratios (with 95% CIs) for mortality associated with heatwaves across different definitions.

**Table S4.** Odds ratios (with 95% CIs) of exposure to heatwaves across different definitions on mortality using daily mean temperature as the temperature metric of heatwave.

**Table S5.** Odds ratios (with 95% CIs) for mortality associated with heatwaves across different definitions with adjustment for PM_2.5_.

**Table S6.** Odds ratios (with 95% CIs) for mortality associated with heatwaves across different definitions with adjustment for PM_10_.

**Table S7.** Odds ratios (with 95% CIs) for mortality associated with heatwaves across different definitions with adjustment for NO_2_.

**Table S8.** Odds ratios (with 95% CIs) for mortality associated with heatwaves across different definitions with adjustment for SO_2_.

**Table S9.** Odds ratios (with 95% CIs) for mortality associated with heatwaves across different definitions with adjustment for CO.

**Table S10.** Lag effect for associations between heatwaves exposure and mortality.

**Table S11.** Cumulative effects of the frequency of heatwave days during lag 0–3 on mortality.

**Table S12.** Odds ratios (with 95% CIs) for heatwave-related mortality using alternative control selection (±14 days around the case day).

**Table S13.** Sensitivity analysis of heatwave-mortality associations using natural cubic spline for relative humidity.

**Table S14.** Associations between different types of heatwaves and mortality excluding the COVID-19 period (2016 to 2019 and 2022).

**Table S15.** Subgroup-specific associations between heatwave exposure and mortality by sex under alternative heatwave definitions (P92.5 and P90).

**Table S16.** Subgroup-specific associations between heatwave exposure and mortality by age group under alternative heatwave definitions (P92.5 and P90).

**Table S17.** Subgroup-specific associations between heatwave exposure and mortality by cause of death under alternative heatwave definitions (P92.5 and P90).


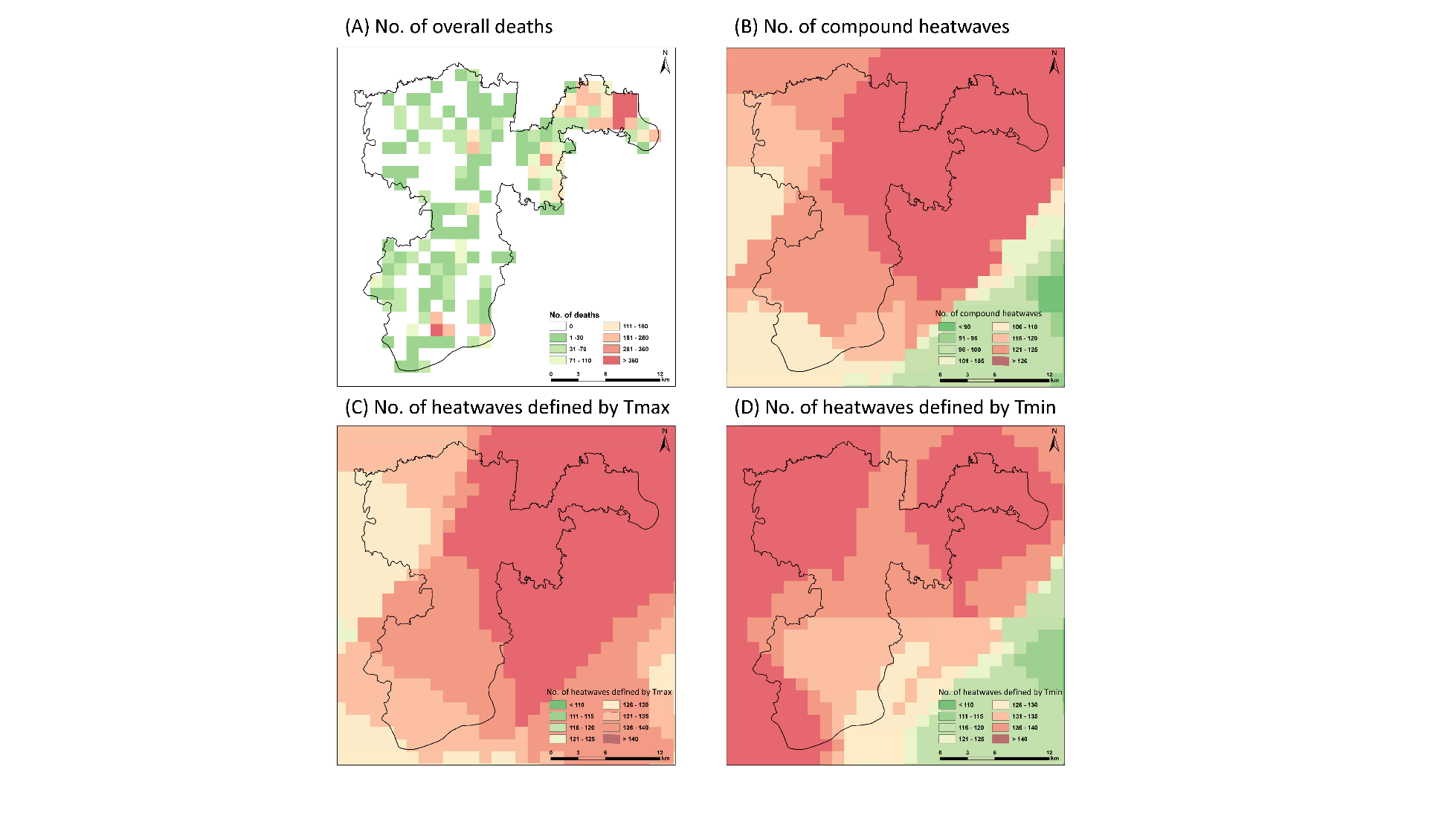


**Figure S1.** Spatial distribution of (A) death cases, (B) compound heatwave, (C) heatwave defined by daily maximum temperature, and (D) heatwave defined by daily minimum temperature from 2016 to 2022.

Abbreviations: T_max_, daily maximum temperature; T_min_, daily minimum temperature.

**Table S1.** Descriptive statistics of heatwave definitions and number of heat wave days on case days and control days from 2016 to 2022.

| Name | Heatwave definitions | *N* (%) | | |
| --- | --- | --- | --- | --- |
|  |  | Compound heatwave days | Heatwave days defined by T_max_ | Heatwave days defined by T_min_ |
| **On case days (n=17,552)** | | | | |
| P90_2d | ≥P90 with ≥2-d duration | 3352 (19.1) | 3982 (22.7) | 4170 (23.8) |
| P90_3d | ≥P90 with ≥3-d duration | 3110 (17.7) | 3560 (20.3) | 3595 (20.5) |
| P90_4d | ≥P90 with ≥4-d duration | 2736 (15.6) | 3218 (18.3) | 3254 (18.5) |
| P92.5_2d | ≥P92.5 with ≥2-d duration | 2560 (14.6) | 3008 (17.1) | 3119 (17.8) |
| P92.5_3d | ≥P92.5 with ≥3-d duration | 2358 (13.4) | 2765 (15.8) | 2778 (15.8) |
| P92.5_4d | ≥P92.5 with ≥4-d duration | 2229 (12.7) | 2537 (14.5) | 2517 (14.3) |
| P95_2d | ≥P95 with ≥2-d duration | 1673 (9.5) | 2177 (12.4) | 2012 (11.5) |
| P95_3d | ≥P95 with ≥3-d duration | 1532 (8.7) | 1954 (11.1) | 1737 (9.9) |
| P95_4d | ≥P95 with ≥4-d duration | 1326 (7.6) | 1784 (10.2) | 1579 (9.0) |
| P97.5_2d | ≥P97.5 with ≥2-d duration | 720 (4.1) | 1093 (6.2) | 942 (5.4) |
| P97.5_3d | ≥P97.5 with ≥3-d duration | 702 (4.0) | 987 (5.6) | 799 (4.6) |
| P97.5_4d | ≥P97.5 with ≥4-d duration | 652 (3.7) | 812 (4.6) | 720 (4.1) |
| **On control days (n=60,131)** | | | | |
| P90_2d | ≥P90 with ≥2-d duration | 10,855 (18.1) | 12,905 (21.5) | 13,812 (23.0) |
| P90_3d | ≥P90 with ≥3-d duration | 10,057 (16.7) | 11,507 (19.1) | 11,764 (19.6) |
| P90_4d | ≥P90 with ≥4-d duration | 8792 (14.6) | 10,460 (17.4) | 10,445 (17.4) |
| P92.5_2d | ≥P92.5 with ≥2-d duration | 8216 (13.7) | 9678 (16.1) | 10,091 (16.8) |
| P92.5_3d | ≥P92.5 with ≥3-d duration | 7574 (12.6) | 8907 (14.8) | 8924 (14.8) |
| P92.5_4d | ≥P92.5 with ≥4-d duration | 7129 (11.9) | 8279 (13.8) | 8016 (13.3) |
| P95_2d | ≥P95 with ≥2-d duration | 5231 (8.7) | 6915 (11.5) | 6352 (10.6) |
| P95_3d | ≥P95 with ≥3-d duration | 4706 (7.8) | 6239 (10.4) | 5424 (9.0) |
| P95_4d | ≥P95 with ≥4-d duration | 4137 (6.9) | 5712 (9.5) | 4966 (8.3) |
| P97.5_2d | ≥P97.5 with ≥2-d duration | 2026 (3.4) | 3302 (5.5) | 2758 (4.6) |
| P97.5_3d | ≥P97.5 with ≥3-d duration | 1968 (3.3) | 2975 (4.9) | 2307 (3.8) |
| P97.5_4d | ≥P97.5 with ≥4-d duration | 1873 (3.1) | 2457 (4.1) | 2065 (3.4) |

Notes: *N* refers to the number of heatwave days on case or control days, and % refers to the proportion of heatwave days to total case or control days; P90, 90th percentiles of the daily temperature distribution; P92.5, 92.5th percentiles of the daily temperature distribution; P95, 95th percentiles of the daily temperature distribution; P97.5, 97.5th percentiles of the daily temperature distribution.

Abbreviations: T_max_, daily maximum temperature; T_min_, daily minimum temperature.

**Table S2.** Summary statistics of ozone and meteorological variables.

| Variable | Mean | SD | Min | Percentiles | | | Max |
| --- | --- | --- | --- | --- | --- | --- | --- |
|  |  |  |  | P_25_ | P_50_ | P_75_ |  |
| Daily minimum temperature, °C | 23.1 | 3.6 | 10.6 | 20.5 | 23.0 | 25.8 | 33.9 |
| Daily maximum temperature, °C | 30.2 | 5.4 | 13.3 | 26.3 | 30.3 | 34.2 | 43.4 |
| Ozone, μg/m^3^ | 108.9 | 40.8 | 17.1 | 79.0 | 107.3 | 135.3 | 258.5 |
| Relative humidity, % | 74.8 | 12.0 | 32.2 | 68.0 | 76.9 | 83.7 | 95.9 |

Abbreviations: SD, standard deviation; Min: minimum; Max: maximum.

**Table S3.** Odds ratios (with 95% CIs) for mortality associated with heatwaves across different definitions.

| Heatwave Definitions | Compound heatwave | |  | Heatwave defined by T_max_ | |  | Heatwave defined by T_min_ | |
| --- | --- | --- | --- | --- | --- | --- | --- | --- |
|  | OR (95% CI) | *P*-value |  | OR (95% CI) | *P*-value |  | OR (95% CI) | *P*-value |
| P90_2d | 1.08 (1.01, 1.15) | 0.019 |  | 1.08 (1.02, 1.15) | 0.012 |  | 1.04 (0.99, 1.10) | 0.144 |
| P90_3d | 1.08 (1.01, 1.15) | 0.017 |  | 1.08 (1.02, 1.15) | 0.014 |  | 1.06 (1.00, 1.12) | 0.070 |
| P90_4d | 1.08 (1.02, 1.15) | 0.012 |  | 1.07 (1.01, 1.15) | 0.028 |  | 1.08 (1.02, 1.15) | 0.006 |
| P92.5_2d | 1.08 (1.01, 1.16) | 0.025 |  | 1.07 (1.00, 1.15) | 0.041 |  | 1.07 (1.01, 1.14) | 0.030 |
| P92.5_3d | 1.08 (1.01, 1.16) | 0.023 |  | 1.07 (1.00, 1.14) | 0.051 |  | 1.09 (1.02, 1.16) | 0.012 |
| P92.5_4d | 1.11 (1.03, 1.19) | 0.005 |  | 1.06 (0.99, 1.13) | 0.119 |  | 1.11 (1.04, 1.19) | 0.001 |
| P95_2d | 1.10 (1.02, 1.20) | 0.013 |  | 1.07 (0.99, 1.15) | 0.075 |  | 1.10 (1.02, 1.18) | 0.011 |
| P95_3d | 1.13 (1.05, 1.23) | 0.002 |  | 1.06 (0.98, 1.14) | 0.119 |  | 1.12 (1.04, 1.21) | 0.004 |
| P95_4d | 1.14 (1.04, 1.25) | 0.005 |  | 1.08 (0.99, 1.16) | 0.068 |  | 1.11 (1.02, 1.20) | 0.012 |
| P97.5_2d | 1.30 (1.15, 1.46) | <0.001 |  | 1.12 (1.02, 1.23) | 0.014 |  | 1.19 (1.08, 1.31) | <0.001 |
| P97.5_3d | 1.32 (1.17, 1.48) | <0.001 |  | 1.13 (1.03, 1.24) | 0.011 |  | 1.24 (1.12, 1.38) | <0.001 |
| P97.5_4d | 1.29 (1.14, 1.46) | <0.001 |  | 1.13 (1.02, 1.26) | 0.019 |  | 1.27 (1.13, 1.43) | <0.001 |

Abbreviations: OR, odds ratio; CI, confidence interval; T_max_, daily maximum temperature; T_min_, daily minimum temperature.

**Table S4.** Odds ratios (with 95% CIs) of exposure to heatwaves across different definitions on mortality using daily mean temperature as the temperature metric of heatwave.

| Heatwave Definitions | OR (95% CI) | *P*-value |
| --- | --- | --- |
| P90_2d | 1.09 (1.02, 1.16) | 0.006 |
| P90_3d | 1.08 (1.02, 1.15) | 0.012 |
| P90_4d | 1.08 (1.02, 1.15) | 0.012 |
| P92.5_2d | 1.07 (1.00, 1.14) | 0.040 |
| P92.5_3d | 1.07 (1.00, 1.14) | 0.053 |
| P92.5_4d | 1.08 (1.01, 1.15) | 0.026 |
| P95_2d | 1.08 (1.01, 1.17) | 0.030 |
| P95_3d | 1.08 (1.00, 1.16) | 0.057 |
| P95_4d | 1.10 (1.01, 1.19) | 0.027 |
| P97.5_2d | 1.18 (1.08, 1.29) | <0.001 |
| P97.5_3d | 1.25 (1.14, 1.38) | <0.001 |
| P97.5_4d | 1.30 (1.17, 1.46) | <0.001 |

Abbreviations: OR, odds ratio; CI, confidence interval.

**Table S5.** Odds ratios (with 95% CIs) for mortality associated with heatwaves across different definitions with adjustment for PM_2.5_.

| Heatwave Definitions | Compound heatwave | |  | Heatwave defined by T_max_ | |  | Heatwave defined by T_min_ | |
| --- | --- | --- | --- | --- | --- | --- | --- | --- |
|  | OR (95% CI) | *P*-value |  | OR (95% CI) | *P*-value |  | OR (95% CI) | *P*-value |
| P90_2d | 1.08 (1.01, 1.15) | 0.020 |  | 1.09 (1.02, 1.16) | 0.010 |  | 1.04 (0.99, 1.10) | 0.129 |
| P90_3d | 1.08 (1.01, 1.15) | 0.016 |  | 1.09 (1.02, 1.16) | 0.011 |  | 1.06 (0.99, 1.12) | 0.077 |
| P90_4d | 1.08 (1.02, 1.16) | 0.012 |  | 1.08 (1.01, 1.15) | 0.022 |  | 1.08 (1.02, 1.15) | 0.008 |
| P92.5_2d | 1.09 (1.02, 1.17) | 0.016 |  | 1.08 (1.01, 1.16) | 0.020 |  | 1.07 (1.00, 1.14) | 0.039 |
| P92.5_3d | 1.09 (1.01, 1.17) | 0.019 |  | 1.08 (1.01, 1.16) | 0.026 |  | 1.08 (1.01, 1.15) | 0.018 |
| P92.5_4d | 1.11 (1.04, 1.19) | 0.003 |  | 1.07 (1.00, 1.14) | 0.063 |  | 1.11 (1.04, 1.19) | 0.001 |
| P95_2d | 1.09 (1.01, 1.18) | 0.029 |  | 1.07 (1.00, 1.16) | 0.059 |  | 1.09 (1.01, 1.17) | 0.022 |
| P95_3d | 1.12 (1.03, 1.21) | 0.007 |  | 1.06 (0.99, 1.15) | 0.112 |  | 1.11 (1.03, 1.20) | 0.009 |
| P95_4d | 1.12 (1.02, 1.22) | 0.016 |  | 1.08 (1.00, 1.16) | 0.060 |  | 1.10 (1.01, 1.19) | 0.024 |
| P97.5_2d | 1.25 (1.11, 1.41) | <0.001 |  | 1.10 (1.01, 1.21) | 0.032 |  | 1.15 (1.05, 1.27) | 0.004 |
| P97.5_3d | 1.27 (1.12, 1.43) | <0.001 |  | 1.11 (1.01, 1.22) | 0.033 |  | 1.20 (1.08, 1.34) | 0.001 |
| P97.5_4d | 1.23 (1.08, 1.40) | 0.002 |  | 1.10 (0.99, 1.23) | 0.071 |  | 1.23 (1.09, 1.38) | 0.001 |

Abbreviations: OR, odds ratio; CI, confidence interval; PM_2.5_, fine particulate matter; T_max_, daily maximum temperature; T_min_, daily minimum temperature.

**Table S6.** Odds ratios (with 95% CIs) for mortality associated with heat waves across different definitions with adjustment for PM_10_.

| Heatwave Definitions | Compound heatwave | |  | Heatwave defined by T_max_ | |  | Heatwave defined by T_min_ | |
| --- | --- | --- | --- | --- | --- | --- | --- | --- |
|  | OR (95% CI) | *P*-value |  | OR (95% CI) | *P*-value |  | OR (95% CI) | *P*-value |
| P90_2d | 1.08 (1.02, 1.15) | 0.012 |  | 1.09 (1.02, 1.16) | 0.007 |  | 1.05 (1.00, 1.11) | 0.074 |
| P90_3d | 1.09 (1.02, 1.16) | 0.010 |  | 1.09 (1.02, 1.16) | 0.008 |  | 1.07 (1.00, 1.13) | 0.036 |
| P90_4d | 1.09 (1.02, 1.16) | 0.007 |  | 1.08 (1.01, 1.15) | 0.017 |  | 1.09 (1.03, 1.16) | 0.003 |
| P92.5_2d | 1.09 (1.02, 1.17) | 0.014 |  | 1.08 (1.01, 1.16) | 0.019 |  | 1.08 (1.01, 1.15) | 0.019 |
| P92.5_3d | 1.09 (1.02, 1.17) | 0.015 |  | 1.08 (1.01, 1.16) | 0.027 |  | 1.09 (1.02, 1.16) | 0.008 |
| P92.5_4d | 1.12 (1.04, 1.20) | 0.003 |  | 1.07 (0.99, 1.14) | 0.070 |  | 1.12 (1.05, 1.20) | 0.001 |
| P95_2d | 1.10 (1.02, 1.19) | 0.019 |  | 1.08 (1.00, 1.16) | 0.053 |  | 1.10 (1.02, 1.18) | 0.013 |
| P95_3d | 1.13 (1.04, 1.22) | 0.004 |  | 1.07 (0.99, 1.15) | 0.101 |  | 1.12 (1.03, 1.21) | 0.005 |
| P95_4d | 1.13 (1.03, 1.24) | 0.009 |  | 1.08 (1.00, 1.17) | 0.055 |  | 1.11 (1.02, 1.20) | 0.016 |
| P97.5_2d | 1.27 (1.13, 1.44) | <0.001 |  | 1.11 (1.01, 1.22) | 0.023 |  | 1.17 (1.06, 1.29) | 0.002 |
| P97.5_3d | 1.29 (1.14, 1.46) | <0.001 |  | 1.12 (1.02, 1.23) | 0.020 |  | 1.22 (1.09, 1.36) | <0.001 |
| P97.5_4d | 1.26 (1.10, 1.43) | 0.001 |  | 1.11 (1.00, 1.24) | 0.049 |  | 1.25 (1.11, 1.40) | <0.001 |

Abbreviations: OR, odds ratio; CI, confidence interval; PM_10_, inhalable particulate matter; T_max_, daily maximum temperature; T_min_, daily minimum temperature.

**Table S7.** Odds ratios (with 95% CIs) for mortality associated with heat waves across different definitions with adjustment for NO_2_.

| Heatwave Definitions | Compound heatwave | |  | Heatwave defined by T_max_ | |  | Heatwave defined by T_min_ | |
| --- | --- | --- | --- | --- | --- | --- | --- | --- |
|  | OR (95% CI) | *P*-value |  | OR (95% CI) | *P*-value |  | OR (95% CI) | *P*-value |
| P90_2d | 1.09 (1.02, 1.16) | 0.008 |  | 1.09 (1.03, 1.16) | 0.006 |  | 1.05 (1.00, 1.11) | 0.068 |
| P90_3d | 1.09 (1.02, 1.16) | 0.007 |  | 1.09 (1.02, 1.16) | 0.008 |  | 1.07 (1.01, 1.13) | 0.033 |
| P90_4d | 1.09 (1.03, 1.17) | 0.006 |  | 1.08 (1.01, 1.15) | 0.018 |  | 1.09 (1.03, 1.16) | 0.003 |
| P92.5_2d | 1.09 (1.02, 1.17) | 0.012 |  | 1.08 (1.01, 1.16) | 0.021 |  | 1.08 (1.02, 1.15) | 0.015 |
| P92.5_3d | 1.09 (1.02, 1.17) | 0.013 |  | 1.08 (1.01, 1.15) | 0.030 |  | 1.09 (1.03, 1.17) | 0.007 |
| P92.5_4d | 1.12 (1.04, 1.20) | 0.003 |  | 1.07 (1.00, 1.14) | 0.067 |  | 1.12 (1.05, 1.20) | 0.001 |
| P95_2d | 1.11 (1.02, 1.20) | 0.012 |  | 1.08 (1.00, 1.16) | 0.051 |  | 1.10 (1.03, 1.19) | 0.009 |
| P95_3d | 1.13 (1.05, 1.23) | 0.002 |  | 1.07 (0.99, 1.15) | 0.085 |  | 1.12 (1.04, 1.21) | 0.003 |
| P95_4d | 1.14 (1.04, 1.24) | 0.005 |  | 1.08 (1.00, 1.17) | 0.051 |  | 1.11 (1.03, 1.21) | 0.010 |
| P97.5_2d | 1.27 (1.12, 1.43) | <0.001 |  | 1.10 (1.01, 1.21) | 0.036 |  | 1.17 (1.06, 1.29) | 0.001 |
| P97.5_3d | 1.28 (1.14, 1.45) | <0.001 |  | 1.11 (1.01, 1.22) | 0.028 |  | 1.22 (1.09, 1.36) | <0.001 |
| P97.5_4d | 1.25 (1.10, 1.42) | <0.001 |  | 1.11 (1.00, 1.24) | 0.056 |  | 1.24 (1.11, 1.40) | <0.001 |

Abbreviations: OR, odds ratio; CI, confidence interval; NO_2_, nitrogen dioxide; T_max_, daily maximum temperature; T_min_, daily minimum temperature.

**Table S8.** Odds ratios (with 95% CIs) for mortality associated with heat waves across different definitions with adjustment for SO_2_.

| Heatwave Definitions | Compound heatwave | |  | Heatwave defined by T_max_ | |  | Heatwave defined by T_min_ | |
| --- | --- | --- | --- | --- | --- | --- | --- | --- |
|  | OR (95% CI) | *P*-value |  | OR (95% CI) | *P*-value |  | OR (95% CI) | *P*-value |
| P90_2d | 1.07 (1.01, 1.14) | 0.029 |  | 1.08 (1.01, 1.15) | 0.017 |  | 1.04 (0.99, 1.10) | 0.152 |
| P90_3d | 1.07 (1.01, 1.15) | 0.028 |  | 1.08 (1.01, 1.15) | 0.023 |  | 1.05 (0.99, 1.12) | 0.086 |
| P90_4d | 1.08 (1.01, 1.15) | 0.022 |  | 1.07 (1.00, 1.14) | 0.046 |  | 1.08 (1.02, 1.15) | 0.009 |
| P92.5_2d | 1.07 (1.00, 1.15) | 0.048 |  | 1.06 (0.99, 1.14) | 0.074 |  | 1.07 (1.00, 1.14) | 0.042 |
| P92.5_3d | 1.08 (1.00, 1.16) | 0.047 |  | 1.06 (0.99, 1.14) | 0.096 |  | 1.08 (1.01, 1.15) | 0.020 |
| P92.5_4d | 1.10 (1.02, 1.18) | 0.011 |  | 1.05 (0.97, 1.12) | 0.212 |  | 1.11 (1.04, 1.18) | 0.003 |
| P95_2d | 1.10 (1.01, 1.19) | 0.026 |  | 1.06 (0.98, 1.14) | 0.130 |  | 1.09 (1.01, 1.18) | 0.019 |
| P95_3d | 1.12 (1.03, 1.22) | 0.006 |  | 1.05 (0.97, 1.14) | 0.199 |  | 1.11 (1.03, 1.20) | 0.009 |
| P95_4d | 1.13 (1.03, 1.24) | 0.011 |  | 1.06 (0.98, 1.15) | 0.125 |  | 1.10 (1.01, 1.20) | 0.025 |
| P97.5_2d | 1.29 (1.15, 1.45) | <0.001 |  | 1.11 (1.02, 1.22) | 0.022 |  | 1.18 (1.07, 1.30) | 0.001 |
| P97.5_3d | 1.31 (1.16, 1.48) | <0.001 |  | 1.12 (1.02, 1.23) | 0.018 |  | 1.23 (1.11, 1.37) | <0.001 |
| P97.5_4d | 1.28 (1.13, 1.46) | <0.001 |  | 1.13 (1.02, 1.26) | 0.022 |  | 1.26 (1.13, 1.42) | <0.001 |

Abbreviations: OR, odds ratio; CI, confidence interval; SO_2_, sulfur dioxide; T_max_, daily maximum temperature; T_min_, daily minimum temperature.

**Table S9.** Odds ratios (with 95% CIs) for mortality associated with heat waves across different definitions with adjustment for CO.

| Heatwave Definitions | Compound heatwave | |  | Heatwave defined by T_max_ | |  | Heatwave defined by T_min_ | |
| --- | --- | --- | --- | --- | --- | --- | --- | --- |
|  | OR (95% CI) | *P*-value |  | OR (95% CI) | *P*-value |  | OR (95% CI) | *P*-value |
| P90_2d | 1.09 (1.02, 1.16) | 0.008 |  | 1.09 (1.03, 1.16) | 0.006 |  | 1.05 (1.00, 1.11) | 0.068 |
| P90_3d | 1.09 (1.02, 1.16) | 0.007 |  | 1.09 (1.02, 1.16) | 0.008 |  | 1.07 (1.01, 1.13) | 0.033 |
| P90_4d | 1.09 (1.03, 1.17) | 0.006 |  | 1.08 (1.01, 1.15) | 0.018 |  | 1.09 (1.03, 1.16) | 0.003 |
| P92.5_2d | 1.09 (1.02, 1.17) | 0.012 |  | 1.08 (1.01, 1.16) | 0.021 |  | 1.08 (1.02, 1.15) | 0.015 |
| P92.5_3d | 1.09 (1.02, 1.17) | 0.013 |  | 1.08 (1.01, 1.15) | 0.030 |  | 1.09 (1.03, 1.17) | 0.007 |
| P92.5_4d | 1.12 (1.04, 1.20) | 0.003 |  | 1.07 (1.00, 1.14) | 0.067 |  | 1.12 (1.05, 1.20) | 0.001 |
| P95_2d | 1.11 (1.02, 1.20) | 0.012 |  | 1.08 (1.00, 1.16) | 0.051 |  | 1.10 (1.03, 1.19) | 0.009 |
| P95_3d | 1.13 (1.05, 1.23) | 0.002 |  | 1.07 (0.99, 1.15) | 0.085 |  | 1.12 (1.04, 1.21) | 0.003 |
| P95_4d | 1.14 (1.04, 1.24) | 0.005 |  | 1.08 (1.00, 1.17) | 0.051 |  | 1.11 (1.03, 1.21) | 0.010 |
| P97.5_2d | 1.27 (1.12, 1.43) | <0.001 |  | 1.10 (1.01, 1.21) | 0.036 |  | 1.17 (1.06, 1.29) | 0.001 |
| P97.5_3d | 1.28 (1.14, 1.45) | <0.001 |  | 1.11 (1.01, 1.22) | 0.028 |  | 1.22 (1.09, 1.36) | <0.001 |
| P97.5_4d | 1.25 (1.10, 1.42) | <0.001 |  | 1.11 (1.00, 1.24) | 0.056 |  | 1.24 (1.11, 1.40) | <0.001 |

Abbreviations: OR, odds ratio; CI, confidence interval; CO, carbon monoxide; T_max_, daily maximum temperature; T_min_, daily minimum temperature.

**Table S10.** Lag effect for associations between heatwaves exposure and mortality.

| Heatwave Definitions | Compound heatwave | |  | Heatwave defined by T_max_ | |  | Heatwave defined by T_min_ | |
| --- | --- | --- | --- | --- | --- | --- | --- | --- |
|  | OR (95% CI) | *P*-value |  | OR (95% CI) | *P*-value |  | OR (95% CI) | *P*-value |
| Lag 0 |  |  |  |  |  |  |  |  |
| P90_2d | 1.08 (1.01, 1.15) | 0.019 |  | 1.08 (1.02, 1.15) | 0.012 |  | 1.04 (0.99, 1.10) | 0.144 |
| P90_3d | 1.08 (1.01, 1.15) | 0.017 |  | 1.08 (1.02, 1.15) | 0.014 |  | 1.06 (1.00, 1.12) | 0.070 |
| P90_4d | 1.08 (1.02, 1.15) | 0.012 |  | 1.07 (1.01, 1.15) | 0.028 |  | 1.08 (1.02, 1.15) | 0.006 |
| P92.5_2d | 1.08 (1.01, 1.16) | 0.025 |  | 1.07 (1.00, 1.15) | 0.041 |  | 1.07 (1.01, 1.14) | 0.030 |
| P92.5_3d | 1.08 (1.01, 1.16) | 0.023 |  | 1.07 (1.00, 1.14) | 0.051 |  | 1.09 (1.02, 1.16) | 0.012 |
| P92.5_4d | 1.11 (1.03, 1.19) | 0.005 |  | 1.06 (0.99, 1.13) | 0.119 |  | 1.11 (1.04, 1.19) | 0.001 |
| P95_2d | 1.10 (1.02, 1.20) | 0.013 |  | 1.07 (0.99, 1.15) | 0.075 |  | 1.10 (1.02, 1.18) | 0.011 |
| P95_3d | 1.13 (1.05, 1.23) | 0.002 |  | 1.06 (0.98, 1.14) | 0.119 |  | 1.12 (1.04, 1.21) | 0.004 |
| P95_4d | 1.14 (1.04, 1.25) | 0.005 |  | 1.08 (0.99, 1.16) | 0.068 |  | 1.11 (1.02, 1.20) | 0.012 |
| P97.5_2d | 1.30 (1.15, 1.46) | <0.001 |  | 1.12 (1.02, 1.23) | 0.014 |  | 1.19 (1.08, 1.31) | <0.001 |
| P97.5_3d | 1.32 (1.17, 1.48) | <0.001 |  | 1.13 (1.03, 1.24) | 0.011 |  | 1.24 (1.12, 1.38) | <0.001 |
| P97.5_4d | 1.29 (1.14, 1.46) | <0.001 |  | 1.13 (1.02, 1.26) | 0.019 |  | 1.27 (1.13, 1.43) | <0.001 |
| Lag 1 |  |  |  |  |  |  |  |  |
| P90_2d | 1.06 (1.00, 1.13) | 0.054 |  | 1.06 (1.00, 1.13) | 0.034 |  | 1.06 (1.00, 1.12) | 0.032 |
| P90_3d | 1.08 (1.01, 1.15) | 0.017 |  | 1.06 (1.00, 1.13) | 0.052 |  | 1.07 (1.01, 1.13) | 0.028 |
| P90_4d | 1.07 (1.00, 1.14) | 0.044 |  | 1.06 (1.00, 1.13) | 0.063 |  | 1.08 (1.02, 1.14) | 0.011 |
| P92.5_2d | 1.12 (1.05, 1.20) | 0.001 |  | 1.08 (1.02, 1.15) | 0.011 |  | 1.12 (1.05, 1.19) | <0.001 |
| P92.5_3d | 1.12 (1.05, 1.20) | 0.001 |  | 1.09 (1.02, 1.16) | 0.010 |  | 1.12 (1.05, 1.19) | <0.001 |
| P92.5_4d | 1.13 (1.05, 1.21) | 0.001 |  | 1.07 (1.01, 1.15) | 0.032 |  | 1.12 (1.05, 1.20) | <0.001 |
| P95_2d | 1.15 (1.07, 1.24) | <0.001 |  | 1.10 (1.03, 1.18) | 0.005 |  | 1.14 (1.07, 1.22) | <0.001 |
| P95_3d | 1.15 (1.06, 1.24) | <0.001 |  | 1.10 (1.03, 1.18) | 0.008 |  | 1.14 (1.06, 1.23) | <0.001 |
| P95_4d | 1.12 (1.03, 1.22) | 0.010 |  | 1.08 (1.00, 1.16) | 0.053 |  | 1.12 (1.04, 1.21) | 0.005 |
| P97.5_2d | 1.33 (1.18, 1.49) | <0.001 |  | 1.17 (1.07, 1.27) | <0.001 |  | 1.26 (1.15, 1.38) | <0.001 |
| P97.5_3d | 1.37 (1.22, 1.54) | <0.001 |  | 1.19 (1.09, 1.31) | <0.001 |  | 1.29 (1.16, 1.44) | <0.001 |
| P97.5_4d | 1.36 (1.20, 1.54) | <0.001 |  | 1.16 (1.05, 1.28) | 0.004 |  | 1.36 (1.21, 1.52) | <0.001 |
| Lag 2 |  |  |  |  |  |  |  |  |
| P90_2d | 1.08 (1.02, 1.15) | 0.005 |  | 1.08 (1.02, 1.14) | 0.008 |  | 1.07 (1.02, 1.13) | 0.007 |
| P90_3d | 1.11 (1.05, 1.18) | <0.001 |  | 1.08 (1.02, 1.14) | 0.012 |  | 1.08 (1.03, 1.15) | 0.005 |
| P90_4d | 1.10 (1.04, 1.17) | 0.002 |  | 1.08 (1.01, 1.14) | 0.017 |  | 1.09 (1.03, 1.16) | 0.002 |
| P92.5_2d | 1.11 (1.05, 1.18) | 0.001 |  | 1.09 (1.02, 1.15) | 0.006 |  | 1.09 (1.03, 1.15) | 0.004 |
| P92.5_3d | 1.14 (1.07, 1.22) | <0.001 |  | 1.10 (1.04, 1.17) | 0.002 |  | 1.12 (1.05, 1.19) | <0.001 |
| P92.5_4d | 1.14 (1.07, 1.22) | <0.001 |  | 1.08 (1.02, 1.15) | 0.012 |  | 1.13 (1.06, 1.20) | <0.001 |
| P95_2d | 1.14 (1.06, 1.22) | <0.001 |  | 1.11 (1.04, 1.19) | 0.001 |  | 1.12 (1.05, 1.19) | 0.001 |
| P95_3d | 1.13 (1.04, 1.21) | 0.002 |  | 1.11 (1.04, 1.19) | 0.002 |  | 1.13 (1.06, 1.22) | <0.001 |
| P95_4d | 1.13 (1.04, 1.22) | 0.006 |  | 1.11 (1.03, 1.19) | 0.006 |  | 1.11 (1.03, 1.20) | 0.005 |
| P97.5_2d | 1.27 (1.14, 1.42) | <0.001 |  | 1.16 (1.07, 1.26) | <0.001 |  | 1.25 (1.14, 1.36) | <0.001 |
| P97.5_3d | 1.28 (1.14, 1.44) | <0.001 |  | 1.18 (1.08, 1.29) | <0.001 |  | 1.28 (1.15, 1.41) | <0.001 |
| P97.5_4d | 1.30 (1.15, 1.46) | <0.001 |  | 1.16 (1.05, 1.28) | 0.003 |  | 1.31 (1.17, 1.46) | <0.001 |
| Lag 3 |  |  |  |  |  |  |  |  |
| P90_2d | 1.09 (1.03, 1.15) | 0.003 |  | 1.07 (1.01, 1.13) | 0.012 |  | 1.11 (1.02, 1.20) | 0.015 |
| P90_3d | 1.09 (1.03, 1.15) | 0.004 |  | 1.08 (1.02, 1.14) | 0.010 |  | 1.13 (1.03, 1.23) | 0.007 |
| P90_4d | 1.10 (1.03, 1.17) | 0.002 |  | 1.10 (1.04, 1.17) | 0.001 |  | 1.12 (1.02, 1.24) | 0.019 |
| P92.5_2d | 1.10 (1.03, 1.16) | 0.003 |  | 1.09 (1.03, 1.16) | 0.004 |  | 1.03 (0.98, 1.08) | 0.274 |
| P92.5_3d | 1.11 (1.04, 1.18) | 0.001 |  | 1.09 (1.03, 1.16) | 0.004 |  | 1.06 (1.00, 1.12) | 0.039 |
| P92.5_4d | 1.11 (1.04, 1.18) | 0.002 |  | 1.09 (1.03, 1.16) | 0.006 |  | 1.07 (1.01, 1.14) | 0.015 |
| P95_2d | 1.07 (1.00, 1.15) | 0.048 |  | 1.09 (1.02, 1.16) | 0.007 |  | 1.08 (1.02, 1.14) | 0.005 |
| P95_3d | 1.08 (1.00, 1.16) | 0.043 |  | 1.09 (1.02, 1.17) | 0.010 |  | 1.10 (1.04, 1.17) | 0.001 |
| P95_4d | 1.06 (0.98, 1.15) | 0.150 |  | 1.07 (1.00, 1.15) | 0.047 |  | 1.12 (1.05, 1.19) | <0.001 |
| P97.5_2d | 1.23 (1.10, 1.37) | <0.001 |  | 1.07 (1.01, 1.13) | 0.012 |  | 1.05 (0.99, 1.12) | 0.129 |
| P97.5_3d | 1.22 (1.09, 1.36) | <0.001 |  | 1.08 (1.02, 1.14) | 0.010 |  | 1.10 (1.03, 1.18) | 0.005 |
| P97.5_4d | 1.22 (1.08, 1.37) | 0.001 |  | 1.10 (1.04, 1.17) | 0.001 |  | 1.09 (1.01, 1.18) | 0.020 |

Abbreviations: OR, odds ratio; CI, confidence interval; T_max_, daily maximum temperature; T_min_, daily minimum temperature.

**Table S11.** Cumulative effects of the frequency of heatwave days during lag 0–3 on mortality.

| Heatwave Definitions | Heatwave Frequency | Compound heatwave | |  | Heatwave defined by T_max_ | |  | Heatwave defined by T_min_ | |
| --- | --- | --- | --- | --- | --- | --- | --- | --- | --- |
|  |  | OR (95% CI) | *P*-value |  | OR (95% CI) | *P*-value |  | OR (95% CI) | *P*-value |
| P90_2d | 1 | 0.99 (0.91, 1.09) | 0.884 |  | 0.99 (0.91, 1.08) | 0.853 |  | 0.93 (0.86, 1.01) | 0.075 |
|  | 2 | 0.95 (0.88, 1.04) | 0.270 |  | 1.01 (0.94, 1.08) | 0.841 |  | 1.02 (0.96, 1.09) | 0.473 |
|  | 3 | 1.03 (0.95, 1.13) | 0.466 |  | 1.01 (0.93, 1.10) | 0.832 |  | 1.00 (0.92, 1.08) | 0.906 |
|  | 4 | 1.14 (1.05, 1.24) | 0.001 |  | 1.15 (1.06, 1.25) | 0.001 |  | 1.09 (1.01, 1.18) | 0.022 |
| P90_3d | 1 | 0.99 (0.89, 1.10) | 0.833 |  | 0.96 (0.86, 1.06) | 0.404 |  | 0.96 (0.87, 1.06) | 0.424 |
|  | 2 | 0.95 (0.86, 1.05) | 0.323 |  | 0.97 (0.89, 1.06) | 0.514 |  | 1.03 (0.94, 1.13) | 0.552 |
|  | 3 | 1.03 (0.95, 1.13) | 0.476 |  | 0.99 (0.91, 1.08) | 0.809 |  | 1.01 (0.93, 1.09) | 0.901 |
|  | 4 | 1.15 (1.06, 1.24) | 0.001 |  | 1.13 (1.05, 1.23) | 0.002 |  | 1.10 (1.02, 1.18) | 0.013 |
| P90_4d | 1 | 1.01 (0.90, 1.13) | 0.893 |  | 0.98 (0.88, 1.09) | 0.721 |  | 1.02 (0.92, 1.13) | 0.712 |
|  | 2 | 0.93 (0.83, 1.05) | 0.230 |  | 0.93 (0.83, 1.03) | 0.173 |  | 1.01 (0.91, 1.11) | 0.909 |
|  | 3 | 0.98 (0.89, 1.08) | 0.705 |  | 0.96 (0.87, 1.06) | 0.420 |  | 1.04 (0.95, 1.15) | 0.393 |
|  | 4 | 1.14 (1.05, 1.23) | 0.001 |  | 1.13 (1.04, 1.22) | 0.003 |  | 1.11 (1.03, 1.19) | 0.005 |
| P92.5_2d | 1 | 0.98 (0.88, 1.08) | 0.624 |  | 0.98 (0.89, 1.08) | 0.619 |  | 0.96 (0.87, 1.05) | 0.325 |
|  | 2 | 0.99 (0.91, 1.08) | 0.779 |  | 0.98 (0.90, 1.07) | 0.683 |  | 1.02 (0.95, 1.10) | 0.574 |
|  | 3 | 1.13 (1.01, 1.25) | 0.027 |  | 1.07 (0.97, 1.18) | 0.167 |  | 1.04 (0.95, 1.13) | 0.445 |
|  | 4 | 1.14 (1.04, 1.25) | 0.003 |  | 1.12 (1.02, 1.22) | 0.012 |  | 1.16 (1.07, 1.27) | <0.001 |
| P92.5_3d | 1 | 1.00 (0.89, 1.11) | 0.931 |  | 0.98 (0.88, 1.09) | 0.739 |  | 0.96 (0.86, 1.07) | 0.486 |
|  | 2 | 0.97 (0.87, 1.08) | 0.559 |  | 0.93 (0.83, 1.03) | 0.164 |  | 1.01 (0.92, 1.12) | 0.813 |
|  | 3 | 1.12 (1.01, 1.24) | 0.035 |  | 1.08 (0.97, 1.19) | 0.149 |  | 1.04 (0.95, 1.14) | 0.343 |
|  | 4 | 1.14 (1.04, 1.24) | 0.003 |  | 1.11 (1.02, 1.20) | 0.018 |  | 1.16 (1.07, 1.26) | <0.001 |
| P92.5_4d | 1 | 0.97 (0.86, 1.10) | 0.668 |  | 0.99 (0.89, 1.11) | 0.916 |  | 0.96 (0.85, 1.08) | 0.478 |
|  | 2 | 0.96 (0.85, 1.08) | 0.481 |  | 0.90 (0.80, 1.01) | 0.073 |  | 1.03 (0.92, 1.15) | 0.592 |
|  | 3 | 1.14 (1.01, 1.27) | 0.029 |  | 1.02 (0.91, 1.14) | 0.793 |  | 1.04 (0.94, 1.16) | 0.459 |
|  | 4 | 1.14 (1.04, 1.24) | 0.004 |  | 1.09 (1.01, 1.19) | 0.034 |  | 1.16 (1.07, 1.26) | <0.001 |
| P95_2d | 1 | 1.01 (0.90, 1.12) | 0.928 |  | 0.98 (0.88, 1.09) | 0.675 |  | 1.00 (0.90, 1.10) | 0.923 |
|  | 2 | 1.06 (0.96, 1.16) | 0.240 |  | 1.00 (0.91, 1.09) | 0.947 |  | 1.07 (0.98, 1.16) | 0.115 |
|  | 3 | 1.08 (0.97, 1.21) | 0.169 |  | 1.08 (0.97, 1.19) | 0.158 |  | 1.08 (0.97, 1.20) | 0.171 |
|  | 4 | 1.18 (1.05, 1.33) | 0.005 |  | 1.15 (1.04, 1.28) | 0.007 |  | 1.15 (1.03, 1.28) | 0.012 |
| P95_3d | 1 | 1.03 (0.91, 1.16) | 0.686 |  | 0.97 (0.87, 1.08) | 0.578 |  | 0.99 (0.88, 1.12) | 0.900 |
|  | 2 | 1.02 (0.91, 1.15) | 0.714 |  | 0.93 (0.83, 1.04) | 0.210 |  | 1.09 (0.97, 1.21) | 0.134 |
|  | 3 | 1.08 (0.96, 1.20) | 0.195 |  | 1.06 (0.95, 1.17) | 0.302 |  | 1.10 (0.99, 1.22) | 0.090 |
|  | 4 | 1.18 (1.05, 1.33) | 0.007 |  | 1.14 (1.02, 1.26) | 0.016 |  | 1.15 (1.03, 1.28) | 0.011 |
| P95_4d | 1 | 0.97 (0.84, 1.14) | 0.745 |  | 0.97 (0.86, 1.10) | 0.638 |  | 0.99 (0.87, 1.14) | 0.934 |
|  | 2 | 1.00 (0.86, 1.16) | 0.990 |  | 0.90 (0.79, 1.02) | 0.108 |  | 1.04 (0.91, 1.18) | 0.572 |
|  | 3 | 1.00 (0.87, 1.14) | 0.961 |  | 1.00 (0.89, 1.12) | 0.974 |  | 1.05 (0.92, 1.18) | 0.479 |
|  | 4 | 1.15 (1.02, 1.30) | 0.024 |  | 1.12 (1.01, 1.24) | 0.037 |  | 1.13 (1.02, 1.26) | 0.024 |
| P97.5_2d | 1 | 1.14 (0.94, 1.38) | 0.176 |  | 0.97 (0.86, 1.10) | 0.682 |  | 1.11 (0.97, 1.26) | 0.117 |
|  | 2 | 1.18 (0.99, 1.41) | 0.069 |  | 1.01 (0.90, 1.13) | 0.903 |  | 1.20 (1.07, 1.35) | 0.002 |
|  | 3 | 1.38 (1.13, 1.67) | 0.001 |  | 1.21 (1.06, 1.38) | 0.004 |  | 1.23 (1.04, 1.46) | 0.017 |
|  | 4 | 1.33 (1.12, 1.60) | 0.002 |  | 1.17 (0.99, 1.37) | 0.060 |  | 1.35 (1.14, 1.59) | <0.001 |
| P97.5_3d | 1 | 1.12 (0.91, 1.38) | 0.270 |  | 0.95 (0.82, 1.10) | 0.492 |  | 1.17 (0.98, 1.39) | 0.088 |
|  | 2 | 1.25 (1.03, 1.52) | 0.026 |  | 1.03 (0.90, 1.17) | 0.705 |  | 1.32 (1.11, 1.58) | 0.002 |
|  | 3 | 1.37 (1.13, 1.67) | 0.001 |  | 1.21 (1.06, 1.38) | 0.005 |  | 1.23 (1.04, 1.46) | 0.016 |
|  | 4 | 1.34 (1.12, 1.60) | 0.001 |  | 1.16 (0.99, 1.36) | 0.062 |  | 1.35 (1.15, 1.60) | <0.001 |
| P97.5_4d | 1 | 1.01 (0.79, 1.28) | 0.955 |  | 0.95 (0.79, 1.14) | 0.567 |  | 1.12 (0.90, 1.40) | 0.295 |
|  | 2 | 1.25 (1.01, 1.55) | 0.043 |  | 1.03 (0.87, 1.23) | 0.697 |  | 1.29 (1.04, 1.59) | 0.019 |
|  | 3 | 1.36 (1.10, 1.68) | 0.005 |  | 1.10 (0.93, 1.31) | 0.264 |  | 1.38 (1.13, 1.69) | 0.002 |
|  | 4 | 1.31 (1.09, 1.56) | 0.003 |  | 1.14 (0.98, 1.34) | 0.095 |  | 1.36 (1.15, 1.61) | <0.001 |

Abbreviations: OR, odds ratio; CI, confidence interval; T_max_, daily maximum temperature; T_min_, daily minimum temperature.

**Table S12.** Odds ratios (with 95% CIs) for heatwave-related mortality using alternative control selection (±14 days around the case day).

| Heatwave Definitions | Compound heatwave | |  | Heatwave defined by T_max_ | |  | Heatwave defined by T_min_ | |
| --- | --- | --- | --- | --- | --- | --- | --- | --- |
|  | OR (95% CI) | *P*-value |  | OR (95% CI) | *P*-value |  | OR (95% CI) | *P*-value |
| P90_2d | 1.10 (1.05, 1.16) | <0.001 |  | 1.11 (1.06, 1.16) | <0.001 |  | 1.08 (1.03, 1.13) | 0.001 |
| P90_3d | 1.09 (1.04, 1.15) | <0.001 |  | 1.09 (1.04, 1.15) | <0.001 |  | 1.09 (1.03, 1.14) | 0.001 |
| P90_4d | 1.10 (1.05, 1.16) | <0.001 |  | 1.10 (1.04, 1.15) | <0.001 |  | 1.09 (1.04, 1.15) | <0.001 |
| P92.5_2d | 1.09 (1.03, 1.15) | 0.002 |  | 1.08 (1.03, 1.13) | 0.003 |  | 1.09 (1.04, 1.15) | <0.001 |
| P92.5_3d | 1.10 (1.04, 1.16) | 0.001 |  | 1.08 (1.02, 1.14) | 0.004 |  | 1.10 (1.04, 1.16) | <0.001 |
| P92.5_4d | 1.11 (1.05, 1.17) | <0.001 |  | 1.08 (1.03, 1.14) | 0.004 |  | 1.12 (1.06, 1.18) | <0.001 |
| P95_2d | 1.11 (1.04, 1.18) | 0.001 |  | 1.11 (1.05, 1.17) | <0.001 |  | 1.08 (1.02, 1.14) | 0.012 |
| P95_3d | 1.13 (1.06, 1.20) | <0.001 |  | 1.11 (1.04, 1.17) | 0.001 |  | 1.10 (1.03, 1.17) | 0.002 |
| P95_4d | 1.13 (1.05, 1.21) | 0.001 |  | 1.12 (1.05, 1.19) | <0.001 |  | 1.10 (1.03, 1.17) | 0.006 |
| P97.5_2d | 1.23 (1.12, 1.36) | <0.001 |  | 1.13 (1.05, 1.21) | 0.001 |  | 1.14 (1.06, 1.24) | 0.001 |
| P97.5_3d | 1.25 (1.13, 1.38) | <0.001 |  | 1.14 (1.05, 1.23) | 0.001 |  | 1.21 (1.10, 1.32) | <0.001 |
| P97.5_4d | 1.23 (1.11, 1.37) | <0.001 |  | 1.16 (1.06, 1.27) | 0.001 |  | 1.22 (1.11, 1.35) | <0.001 |

Abbreviations: OR, odds ratio; CI, confidence interval; T_max_, daily maximum temperature; T_min_, daily minimum temperature.

**Table S13.** Sensitivity analysis of heatwave-mortality associations using natural cubic spline for relative humidity.

| Heatwave Definitions | Compound heatwave | |  | Heatwave defined by T_max_ | |  | Heatwave defined by T_min_ | |
| --- | --- | --- | --- | --- | --- | --- | --- | --- |
|  | OR (95% CI) | *P*-value |  | OR (95% CI) | *P*-value |  | OR (95% CI) | *P*-value |
| P90_2d | 1.06 (0.99, 1.13) | 0.074 |  | 1.07 (1.00, 1.14) | 0.043 |  | 1.04 (0.98, 1.09) | 0.223 |
| P90_3d | 1.06 (0.99, 1.13) | 0.075 |  | 1.07 (1.00, 1.14) | 0.056 |  | 1.04 (0.98, 1.11) | 0.168 |
| P90_4d | 1.07 (1.00, 1.14) | 0.055 |  | 1.05 (0.99, 1.13) | 0.119 |  | 1.07 (1.01, 1.14) | 0.019 |
| P92.5_2d | 1.05 (0.98, 1.13) | 0.164 |  | 1.04 (0.97, 1.12) | 0.219 |  | 1.05 (0.99, 1.12) | 0.110 |
| P92.5_3d | 1.06 (0.98, 1.14) | 0.150 |  | 1.04 (0.97, 1.12) | 0.290 |  | 1.07 (1.00, 1.14) | 0.055 |
| P92.5_4d | 1.08 (1.00, 1.16) | 0.043 |  | 1.02 (0.95, 1.10) | 0.558 |  | 1.09 (1.02, 1.17) | 0.009 |
| P95_2d | 1.06 (0.97, 1.16) | 0.181 |  | 1.02 (0.94, 1.11) | 0.613 |  | 1.07 (0.99, 1.15) | 0.099 |
| P95_3d | 1.09 (1.00, 1.20) | 0.048 |  | 1.01 (0.93, 1.10) | 0.860 |  | 1.09 (1.00, 1.18) | 0.048 |
| P95_4d | 1.09 (0.98, 1.20) | 0.101 |  | 1.02 (0.94, 1.11) | 0.641 |  | 1.07 (0.98, 1.17) | 0.127 |
| P97.5_2d | 1.25 (1.09, 1.43) | 0.001 |  | 1.05 (0.95, 1.17) | 0.329 |  | 1.14 (1.02, 1.27) | 0.018 |
| P97.5_3d | 1.27 (1.11, 1.46) | 0.001 |  | 1.06 (0.95, 1.19) | 0.275 |  | 1.19 (1.06, 1.35) | 0.004 |
| P97.5_4d | 1.23 (1.07, 1.42) | 0.004 |  | 1.06 (0.94, 1.20) | 0.369 |  | 1.22 (1.07, 1.39) | 0.003 |

Abbreviations: OR, odds ratio; CI, confidence interval; T_max_, daily maximum temperature; T_min_, daily minimum temperature.

**Table S14.** Associations between different types of heatwaves and mortality excluding the COVID-19 period (2016 to 2019 and 2022).

| Heatwave Definitions | Compound heatwave | |  | Heatwave defined by T_max_ | |  | Heatwave defined by T_min_ | |
| --- | --- | --- | --- | --- | --- | --- | --- | --- |
|  | OR (95% CI) | *P*-value |  | OR (95% CI) | *P*-value |  | OR (95% CI) | *P*-value |
| P90_2d | 1.04 (0.97, 1.12) | 0.271 |  | 1.06 (0.99, 1.14) | 0.094 |  | 1.02 (0.96, 1.09) | 0.485 |
| P90_3d | 1.06 (0.98, 1.14) | 0.144 |  | 1.07 (0.99, 1.15) | 0.088 |  | 1.04 (0.97, 1.11) | 0.320 |
| P90_4d | 1.04 (0.97, 1.12) | 0.283 |  | 1.05 (0.98, 1.13) | 0.159 |  | 1.05 (0.98, 1.12) | 0.170 |
| P92.5_2d | 1.07 (0.99, 1.15) | 0.107 |  | 1.09 (1.01, 1.17) | 0.025 |  | 1.04 (0.96, 1.12) | 0.328 |
| P92.5_3d | 1.07 (0.99, 1.16) | 0.083 |  | 1.10 (1.02, 1.18) | 0.017 |  | 1.05 (0.98, 1.13) | 0.169 |
| P92.5_4d | 1.09 (1.01, 1.18) | 0.030 |  | 1.09 (1.01, 1.17) | 0.031 |  | 1.08 (1.00, 1.16) | 0.056 |
| P95_2d | 1.10 (1.00, 1.20) | 0.040 |  | 1.07 (0.99, 1.16) | 0.094 |  | 1.07 (0.98, 1.16) | 0.125 |
| P95_3d | 1.13 (1.03, 1.24) | 0.008 |  | 1.07 (0.98, 1.16) | 0.116 |  | 1.11 (1.02, 1.20) | 0.021 |
| P95_4d | 1.13 (1.02, 1.24) | 0.014 |  | 1.08 (0.99, 1.18) | 0.069 |  | 1.09 (0.99, 1.19) | 0.066 |
| P97.5_2d | 1.28 (1.14, 1.45) | <0.001 |  | 1.16 (1.05, 1.29) | 0.004 |  | 1.20 (1.08, 1.33) | 0.001 |
| P97.5_3d | 1.30 (1.15, 1.47) | <0.001 |  | 1.19 (1.07, 1.32) | 0.002 |  | 1.25 (1.11, 1.39) | <0.001 |
| P97.5_4d | 1.27 (1.12, 1.45) | <0.001 |  | 1.22 (1.09, 1.38) | 0.001 |  | 1.26 (1.12, 1.41) | <0.001 |

Abbreviations: OR, odds ratio; CI, confidence interval; T_max_, daily maximum temperature; T_min_, daily minimum temperature.

**Table S15.** Subgroup-specific associations between heatwave exposure and mortality by sex under alternative heatwave definitions (P92.5 and P90).

| Heatwave Definitions | Man | |  | Woman | |
| --- | --- | --- | --- | --- | --- |
|  | OR (95% CI) | *P*-value |  | OR (95% CI) | *P*-value |
| Compound heatwave | | | | | |
| P90_2d | 1.11 (1.02, 1.21) | 0.012 |  | 1.03 (0.94, 1.14) | 0.524 |
| P90_3d | 1.10 (1.01, 1.19) | 0.024 |  | 1.05 (0.95, 1.16) | 0.320 |
| P90_4d | 1.13 (1.04, 1.23) | 0.003 |  | 1.02 (0.92, 1.12) | 0.748 |
| P92.5_2d | 1.10 (1.01, 1.21) | 0.031 |  | 1.05 (0.94, 1.17) | 0.384 |
| P92.5_3d | 1.12 (1.02, 1.23) | 0.015 |  | 1.03 (0.93, 1.15) | 0.541 |
| P92.5_4d | 1.17 (1.07, 1.28) | 0.001 |  | 1.02 (0.91, 1.14) | 0.713 |
| Heatwave defined by daily maximum temperature | | | | | |
| P90_2d | 1.12 (1.03, 1.21) | 0.008 |  | 1.04 (0.94, 1.14) | 0.457 |
| P90_3d | 1.11 (1.03, 1.21) | 0.010 |  | 1.04 (0.94, 1.15) | 0.470 |
| P90_4d | 1.12 (1.03, 1.21) | 0.009 |  | 1.02 (0.92, 1.12) | 0.766 |
| P92.5_2d | 1.09 (1.00, 1.19) | 0.045 |  | 1.04 (0.94, 1.15) | 0.454 |
| P92.5_3d | 1.09 (0.99, 1.19) | 0.068 |  | 1.05 (0.94, 1.16) | 0.414 |
| P92.5_4d | 1.09 (1.00, 1.19) | 0.058 |  | 1.01 (0.91, 1.12) | 0.903 |
| Heatwave defined by daily minimum temperature | | | | | |
| P90_2d | 1.10 (1.02, 1.18) | 0.010 |  | 0.96 (0.88, 1.05) | 0.412 |
| P90_3d | 1.10 (1.02, 1.19) | 0.012 |  | 0.99 (0.90, 1.09) | 0.841 |
| P90_4d | 1.14 (1.05, 1.23) | 0.001 |  | 1.01 (0.92, 1.11) | 0.796 |
| P92.5_2d | 1.11 (1.02, 1.21) | 0.011 |  | 1.02 (0.92, 1.12) | 0.724 |
| P92.5_3d | 1.12 (1.03, 1.22) | 0.007 |  | 1.04 (0.94, 1.15) | 0.494 |
| P92.5_4d | 1.18 (1.09, 1.29) | <0.001 |  | 1.02 (0.92, 1.13) | 0.663 |

Abbreviations: OR, odds ratio; CI, confidence interval.

**Table S16.** Subgroup-specific associations between heatwave exposure and mortality by age group under alternative heatwave definitions (P92.5 and P90).

| Heatwave Definitions | <75 yrs | |  | ≥75 yrs | |
| --- | --- | --- | --- | --- | --- |
|  | OR (95% CI) | *P*-value |  | OR (95% CI) | *P*-value |
| Compound heatwave | | | | | |
| P90_2d | 1.08 (0.98, 1.18) | 0.120 |  | 1.08 (0.99, 1.18) | 0.076 |
| P90_3d | 1.07 (0.97, 1.17) | 0.157 |  | 1.09 (1.00, 1.19) | 0.048 |
| P90_4d | 1.09 (1.00, 1.20) | 0.063 |  | 1.08 (0.99, 1.18) | 0.091 |
| P92.5_2d | 1.08 (0.98, 1.20) | 0.111 |  | 1.08 (0.98, 1.18) | 0.120 |
| P92.5_3d | 1.10 (0.99, 1.22) | 0.067 |  | 1.07 (0.97, 1.18) | 0.167 |
| P92.5_4d | 1.14 (1.03, 1.26) | 0.013 |  | 1.08 (0.98, 1.19) | 0.130 |
| Heatwave defined by daily maximum temperature | | | | | |
| P90_2d | 1.06 (0.96, 1.16) | 0.235 |  | 1.11 (1.02, 1.21) | 0.018 |
| P90_3d | 1.08 (0.99, 1.19) | 0.096 |  | 1.09 (0.99, 1.19) | 0.068 |
| P90_4d | 1.08 (0.98, 1.18) | 0.117 |  | 1.07 (0.98, 1.17) | 0.118 |
| P92.5_2d | 1.05 (0.95, 1.16) | 0.308 |  | 1.09 (0.99, 1.19) | 0.066 |
| P92.5_3d | 1.07 (0.97, 1.18) | 0.198 |  | 1.07 (0.98, 1.18) | 0.142 |
| P92.5_4d | 1.05 (0.95, 1.16) | 0.300 |  | 1.06 (0.96, 1.16) | 0.247 |
| Heatwave defined by daily minimum temperature | | | | | |
| P90_2d | 1.09 (1.00, 1.19) | 0.053 |  | 1.02 (0.94, 1.10) | 0.664 |
| P90_3d | 1.11 (1.02, 1.20) | 0.020 |  | 1.03 (0.95, 1.12) | 0.502 |
| P90_4d | 1.07 (0.98, 1.17) | 0.147 |  | 1.07 (0.98, 1.15) | 0.121 |
| P92.5_2d | 1.07 (0.98, 1.18) | 0.138 |  | 1.07 (0.99, 1.17) | 0.105 |
| P92.5_3d | 1.14 (1.04, 1.26) | 0.006 |  | 1.10 (1.01, 1.20) | 0.038 |
| P92.5_4d | 1.04 (0.93, 1.16) | 0.468 |  | 1.09 (0.99, 1.19) | 0.068 |

Abbreviations: OR, odds ratio; CI, confidence interval.

**Table S17.** Subgroup-specific associations between heatwave exposure and mortality by cause of death under alternative heatwave definitions (P92.5 and P90).

| Heatwave Definitions | Cardiovascular diseases | |  | Coronary heart disease | |  | Stroke | |  | Hemorrhagic stroke | |  | Ischemic stroke | |  | Respiratory diseases | |  | COPD | |
| --- | --- | --- | --- | --- | --- | --- | --- | --- | --- | --- | --- | --- | --- | --- | --- | --- | --- | --- | --- | --- |
|  | OR (95% CI) | *P*-value |  | OR (95% CI) | *P*-value |  | OR (95% CI) | *P*-value |  | OR (95% CI) | *P*-value |  | OR (95% CI) | *P*-value |  | OR (95% CI) | *P*-value |  | OR (95% CI) | *P*-value |
| Compound heatwave | | | | | | | | | | | | | | | | | | | | |
| P90_2d | 1.07 (0.97, 1.18) | 0.187 |  | 1.24 (1.07, 1.42) | 0.003 |  | 0.79 (0.67, 0.93) | 0.006 |  | 0.79 (0.60, 1.04) | 0.093 |  | 0.80 (0.61, 1.04) | 0.098 |  | 0.94 (0.80, 1.11) | 0.453 |  | 0.94 (0.75, 1.18) | 0.580 |
| P90_3d | 1.08 (0.98, 1.20) | 0.122 |  | 1.24 (1.07, 1.43) | 0.003 |  | 0.84 (0.71, 0.99) | 0.041 |  | 0.84 (0.64, 1.11) | 0.220 |  | 0.84 (0.64, 1.11) | 0.224 |  | 0.98 (0.83, 1.15) | 0.810 |  | 1.02 (0.81, 1.28) | 0.870 |
| P90_4d | 1.10 (1.00, 1.22) | 0.058 |  | 1.23 (1.07, 1.42) | 0.004 |  | 0.90 (0.76, 1.07) | 0.240 |  | 0.89 (0.67, 1.16) | 0.382 |  | 0.95 (0.72, 1.24) | 0.701 |  | 1.02 (0.87, 1.21) | 0.781 |  | 1.06 (0.85, 1.34) | 0.589 |
| P92.5_2d | 1.11 (1.00, 1.24) | 0.056 |  | 1.30 (1.12, 1.52) | 0.001 |  | 0.83 (0.69, 0.99) | 0.043 |  | 0.84 (0.63, 1.12) | 0.237 |  | 0.92 (0.69, 1.24) | 0.588 |  | 0.97 (0.82, 1.16) | 0.769 |  | 1.02 (0.80, 1.30) | 0.877 |
| P92.5_3d | 1.11 (0.99, 1.24) | 0.074 |  | 1.26 (1.08, 1.48) | 0.004 |  | 0.87 (0.73, 1.05) | 0.150 |  | 0.90 (0.67, 1.22) | 0.502 |  | 0.99 (0.74, 1.34) | 0.952 |  | 1.06 (0.88, 1.26) | 0.556 |  | 1.17 (0.91, 1.50) | 0.224 |
| P92.5_4d | 1.15 (1.03, 1.29) | 0.012 |  | 1.35 (1.15, 1.58) | <0.001 |  | 0.88 (0.73, 1.06) | 0.171 |  | 0.89 (0.66, 1.20) | 0.464 |  | 1.05 (0.78, 1.42) | 0.741 |  | 1.07 (0.89, 1.28) | 0.487 |  | 1.14 (0.89, 1.47) | 0.294 |
| Heatwave defined by daily maximum temperature | | | | | | | | | | | | | | | | | | | | |
| P90_2d | 1.08 (0.98, 1.19) | 0.124 |  | 1.22 (1.06, 1.40) | 0.006 |  | 0.81 (0.69, 0.96) | 0.012 |  | 0.77 (0.59, 1.01) | 0.059 |  | 0.81 (0.62, 1.06) | 0.129 |  | 0.95 (0.81, 1.12) | 0.572 |  | 0.95 (0.76, 1.19) | 0.654 |
| P90_3d | 1.08 (0.97, 1.19) | 0.152 |  | 1.22 (1.06, 1.41) | 0.007 |  | 0.84 (0.71, 0.99) | 0.036 |  | 0.82 (0.63, 1.08) | 0.153 |  | 0.86 (0.65, 1.12) | 0.261 |  | 0.98 (0.83, 1.15) | 0.775 |  | 1.02 (0.81, 1.28) | 0.870 |
| P90_4d | 1.09 (0.99, 1.21) | 0.092 |  | 1.24 (1.08, 1.44) | 0.003 |  | 0.84 (0.71, 1.00) | 0.047 |  | 0.89 (0.68, 1.17) | 0.412 |  | 0.87 (0.66, 1.15) | 0.326 |  | 1.00 (0.84, 1.18) | 0.959 |  | 1.06 (0.84, 1.33) | 0.613 |
| P92.5_2d | 1.07 (0.96, 1.19) | 0.217 |  | 1.28 (1.10, 1.49) | 0.001 |  | 0.75 (0.63, 0.90) | 0.002 |  | 0.77 (0.58, 1.03) | 0.078 |  | 0.88 (0.66, 1.16) | 0.362 |  | 0.98 (0.83, 1.17) | 0.847 |  | 1.02 (0.81, 1.30) | 0.846 |
| P92.5_3d | 1.07 (0.96, 1.19) | 0.249 |  | 1.24 (1.06, 1.44) | 0.007 |  | 0.81 (0.67, 0.96) | 0.018 |  | 0.87 (0.65, 1.16) | 0.353 |  | 0.93 (0.70, 1.24) | 0.623 |  | 1.06 (0.89, 1.26) | 0.521 |  | 1.16 (0.91, 1.47) | 0.230 |
| P92.5_4d | 1.09 (0.97, 1.21) | 0.137 |  | 1.27 (1.09, 1.48) | 0.003 |  | 0.81 (0.68, 0.97) | 0.024 |  | 0.91 (0.68, 1.21) | 0.498 |  | 0.95 (0.71, 1.27) | 0.724 |  | 1.02 (0.86, 1.21) | 0.830 |  | 1.12 (0.88, 1.42) | 0.375 |
| Heatwave defined by daily minimum temperature | | | | | | | | | | | | | | | | | | | | |
| P90_2d | 0.99 (0.91, 1.08) | 0.836 |  | 1.02 (0.90, 1.16) | 0.699 |  | 0.90 (0.78, 1.04) | 0.157 |  | 0.86 (0.67, 1.09) | 0.205 |  | 0.84 (0.67, 1.07) | 0.157 |  | 0.94 (0.82, 1.09) | 0.414 |  | 0.99 (0.82, 1.21) | 0.954 |
| P90_3d | 1.01 (0.92, 1.11) | 0.796 |  | 1.12 (0.98, 1.28) | 0.098 |  | 0.81 (0.69, 0.95) | 0.009 |  | 0.81 (0.63, 1.04) | 0.100 |  | 0.79 (0.61, 1.02) | 0.066 |  | 0.97 (0.83, 1.13) | 0.680 |  | 1.03 (0.83, 1.26) | 0.811 |
| P90_4d | 1.06 (0.97, 1.17) | 0.194 |  | 1.17 (1.03, 1.33) | 0.017 |  | 0.89 (0.77, 1.04) | 0.153 |  | 0.86 (0.67, 1.11) | 0.252 |  | 0.89 (0.69, 1.14) | 0.346 |  | 1.02 (0.88, 1.19) | 0.801 |  | 1.13 (0.92, 1.39) | 0.243 |
| P92.5_2d | 1.09 (0.98, 1.20) | 0.102 |  | 1.23 (1.07, 1.42) | 0.004 |  | 0.85 (0.72, 1.00) | 0.057 |  | 0.81 (0.62, 1.06) | 0.130 |  | 0.81 (0.61, 1.06) | 0.125 |  | 1.00 (0.85, 1.18) | 0.970 |  | 0.99 (0.79, 1.23) | 0.902 |
| P92.5_3d | 1.10 (0.99, 1.22) | 0.067 |  | 1.25 (1.08, 1.45) | 0.003 |  | 0.89 (0.75, 1.05) | 0.163 |  | 0.90 (0.68, 1.19) | 0.454 |  | 0.93 (0.70, 1.22) | 0.602 |  | 1.06 (0.90, 1.25) | 0.508 |  | 1.16 (0.92, 1.46) | 0.213 |
| P92.5_4d | 1.17 (1.05, 1.30) | 0.003 |  | 1.35 (1.17, 1.57) | <0.001 |  | 0.92 (0.77, 1.09) | 0.334 |  | 0.89 (0.67, 1.17) | 0.403 |  | 1.07 (0.80, 1.42) | 0.652 |  | 1.04 (0.88, 1.24) | 0.628 |  | 1.06 (0.83, 1.34) | 0.648 |

Abbreviations: OR, odds ratio; CI, confidence interval; COPD, chronic obstructive pulmonary disease.
